# Morphological, Genetic and Biological Evidences to Understand *Meromacrus* Rondani Diversity: New Species and Early Stages (Diptera: Syrphidae)

**DOI:** 10.3390/insects11110791

**Published:** 2020-11-12

**Authors:** Antonio Ricarte, Gabriel J. Souba-Dols, Jeffrey H. Skevington, Javier Quinto, Mª Ángeles Marcos García

**Affiliations:** 1 CIBIO Research Institute (Centro Iberoamericano de la Biodiversidad), Universidad de Alicante, Carretera San Vicente s/n, 03690 San Vicente del Raspeig, Alicante, Spain; gjs.dols@gmail.com (G.J.S.-D.); marcos@ua.es (M.Á.M.G.); 2 Canadian National Collection of Insects, Arachnids and Nematodes, Agriculture and Agri-Food Canada, 960 Carling Avenue, K.W. Neatby Building, Ottawa, ON K1A 0C6, Canada; jhskevington@gmail.com; 3 Instituto de Investigación y Formación Agraria, Pesquera, Alimentaria y de la Producción Ecológica (IFAPA), Centro Málaga (Churriana), s/n, 29140 Cortijo de la Cruz, Málaga, Spain; javier.quinto@juntadeandalucia.es; 4 Departamento de Ecología Tropical, Universidad Autónoma de Yucatán (UADY), Km. 15.5 Carretera de Mérida-X’matkuil, Mérida, Yucatán 4-116, Mexico

**Keywords:** DNA analysis, identification key, male genitalia, neotropical syrphids, puparia, SEM imaging

## Abstract

**Simple Summary:**

Hoverflies or flower flies, technically known as syrphids, are insects providing important ecosystem services. They are used as indicators of biodiversity and habitat quality, as well as models for evolution studies. The better syrphids are known the better can be used with different aims. *Meromacrus* is a genus of showy syrphids that pollinate plants and decompose organic materials in the Americas. However, their classification and biology are still being investigated. In this study, morphology and DNA were used in combination to define species concepts. Two species new to science were discovered, one from Mexico and the other from Peru. The immatures (e.g., larvae or pupae) and breeding sites of these species were also described, with the larva of the Peruvian species happening to be the first ever found in a rotting cactus. To assist those working with immatures, we provide here an identification key to *Meromacrus* species. Our work represents the starting point for a modern revision of the *Meromacrus* classification and provides data that, in future, can be used to interpret evolutionary relationships within this genus.

**Abstract:**

*Meromacrus* is a genus of conspicuous syrphids with saprophagous larvae, ranging from the southern United States to Argentina and Chile. However, this genus is in need of a taxonomic revision. Adults reared from larvae collected in Mexico and Peru, and other material available at different institutional collections were examined. *Meromacrus cactorum* sp. nov., from Peru, *Meromacrus yucatense* sp. nov., from Mexico, their puparia and breeding sites were described. A key to *Meromacrus* puparia is provided. The holotypes of *Meromacrus canusium*, *Meromacrus gloriosus*, *Meromacrus laconicus* and *Meromacrus melmoth* were also examined. The name *Meromacrus draco* is proposed as a junior synonym of *M. gloriosus*. Larvae of *M. cactorum* sp. nov. were found in decaying columnar cacti in Peru, while those of *M. yucatense* sp. nov. in a rot-hole of a *Ceiba pentandra* stump. Obtained results on both taxonomy and biology of these species serve as a first step towards a revision of the entire genus.

## 1. Introduction

Syrphids of the genus *Meromacrus* Rondani, 1848 (Syrphidae: Eristalinae) are large-sized flies that can be distinguished from the other Neotropical eristaline genera by their bare eyes, strongly sinuate vein R_4 + 5_ and tomentose maculae [1]. The 43 species of the genus range from the southern United States to northern Argentina and Chile in the neotropics, with the exception of the presumably erroneous type localities given for *Meromacrus maculatus* (Macquart, 1850) [junior synonym of *Meromacrus acutus* (Fabricius, 1805)] and *Meromacrus canusium* (Walker, 1860) as ‘Afrique’ and the ‘Cape of Good Hope’ (Africa), respectively [2,3,4,5]. The taxonomic status of some species is uncertain. For example, Blatch et al. [4] suggest that *Meromacrus croceatus* Hull, 1960, *Meromacrus draco* Hull, 1942 and *Meromacrus gloriosus* Hull, 1941 might be junior synonyms of *M. canusium*; *Meromacrus cingulatus* Sack, 1920 and *Meromacrus simplex* (Schiner, 1868) were presented in Thompson et al. [2] as synonyms of *Meromacrus nectarinoides* (Lynch Arribálzaga, 1892) and *Meromacrus pachypus* (Wiedemann, 1830), respectively, but without evidence supporting these nomenclatural acts.

Although molecular evidence has proven useful to resolve taxonomic problems in the eristalines e.g. [6,7], current species concepts in *Meromacrus* are based only on classic morphology of adults and their phylogenetic relationships are unknown. The study of the immature stages of syrphids not only provide an important set of characters on which to build more robust phylogenies [8] but is also critical to understand the species requirements in different ecosystems. The typical long-tailed larvae of *Meromacrus* syrphids are saprophagous in rot holes of various tree species, water pockets in banana leaf axils [4,9], bromeliads [10] and *Heliconia* L. flower bracts [11], in decaying banana stems [4,12] and coffee pulp [9]. Larvae of different *Meromacrus* species can be found coexisting in the same tree hole [4]. The larvae/puparia of only six species are described [9]. Adult flies are frequent flower visitors in natural environments [11], but also seem to play an important role in the pollination of some cultivated plants such as *Meromacrus melansoni* Blatch in Blatch et al. [4] in mango trees (Anacardiaceae) [4,13].

The aim of the present study is to stablish the bases for a future revision of the taxonomy and phylogeny of the genus *Meromacrus*. The specific objectives are: (a) to describe new species and address some pending taxonomic problems, (b) to describe new puparia and breeding sites, (c) to update the existing early stage identification key, (d) to explore the molecular support of some species, based on COI (cytochrome *c* oxidase I mitochondrial gene).

## 2. Materials and Methods

### 2.1. Fieldwork and Rearing of Early Stages

In Yucatan (Mexico), *Meromacrus* larvae were collected from a rot hole in a *Ceiba pentandra* (L.) Gaertn. tree (Malvaceae) by Javier Quinto. Larvae were reared by placing them in a plastic cage of 30 × 20 × 8 cm containing part of the original rot-hole substrate (water and woody debris). This cage was placed inside another of 45 × 37 × 24 cm covered by a mesh and filled with a thin layer of dry small woody material from the forest soil of the collection site, to facilitate pupation of larvae. This dry layer was checked daily for puparia. Puparia were marked with a label and after 4 days, when the pupal spiracles protruded entirely, they were individualized in separate small plastic pots. Emerged adults were allowed to die in their pots. The date of pupation and the date of adult emergence were recorded for each specimen. Larvae and puparia were reared under environmental conditions. Larvae were collected on 15 March 2014 and they all pupated between 18 March and 8 April, i.e., a range of 22 days of pupation from the date of the larva finding. The pupal stage lasted 6–31 days and all adults emerged between 25 March and 26 April.

In Trujillo (Peru), larvae were collected in the decaying columnar cacti *Espostoa melanostele* (Vaupel) Borg (Cactaceae) by M. Ángeles Marcos-García on 17 January 2005, and then reared in plastic cages of 24 × 20 × 34 cm containing part of the original substrate where the larvae were found. Cages were covered with a mesh to allow the entrance of oxygen. They were reared under environmental conditions in shade. Puparia were individualized in Petri dishes until adult emergence. Adults reared from larvae collected in Mexico and Peru were dry pinned and their puparia stored in plastic capsules attached to the pin or in a different pin next to the correspondent adult and properly labelled.

### 2.2. Morphological Study

The material examined originates from Costa Rica, Cuba, Mexico, Peru and the USA. Most specimens from Mexico and all from Peru were reared from larvae as stated above. For their morphological study, puparia were cleaned with a fine paint brush after soaking in distilled water following the protocol in Ricarte et al. [14]. Size and shape of the new species’ puparia were studied and measured with a M205C stereo microscope (Leica Microsystems, Heerbrugg, Switzerland) and the software Leica Application Suite v.4.8 (Leica Microsystems [Schweiz] AG, Heerbrugg, Switzerland). Puparium length was measured from the anterior margin to the anus in ventral view. Height and width were measured at their maxima. Width of the posterior respiratory process (PRP) was measured to the end of the tube, at their maxima. Ornaments of the anterior spiracles (AS), pupal spiracles (PS) and PRP were described using a S-3000N scanning electron microscope (SEM, Hitachi, Tokyo, Japan). Head skeletons, also known as cephalopharyngeal skeletons, were extracted with pins from puparia after 30 min soaking in potassium hydroxide (KOH) solution. Then, head skeletons were studied and preserved in glycerine. Drawings of the head skeletons were made from high-resolution photographs taken with a Leica DFC 450 camera (Leica Microsystems [Switzerland] Ltd., Heerbrugg, Switzerland) attached to Leica M205 C stereo microscope (same item as before) using Leica Application Suite X (LAS X) ^®^, v. 3.0.4.16529 software (Leica Microsystems CMS, GmbH, Wetzlar, Germany). For puparia, morphological terminology follows Hartley [15] and Rotheray [16]. Head skeleton terminology follows Hartley [17], Rotheray [18] and Rotheray and Gilbert [19]. The key provided to the *Meromacrus* puparia is adapted from that in Pérez-Bañón et al. [9].

Adults were identified using keys and descriptions in Hull [20], Thompson [3] and Blatch et al. [4] by Antonio Ricarte and M. Ángeles Marcos-García, unless otherwise stated. For adult descriptions, body length was determined by measuring the distance between the apices of the frontal prominence on the head and the abdomen. Male genitalia were examined by relaxing specimens and removing genitalia with an entomological pin. They were cleared in a hot solution of KOH for up to 5 min, immersed in acetic acid to remove excess KOH, washed in 70% alcohol, and stored in microvials containing glycerine. The morphological terminology follows that of Thompson [1]. Species were illustrated with photos, except for the male genitalia that were drawn. Photos were produced as stacks of individual images made with a camera (Leica DFC 450) attached to a binocular stereomicroscope (Leica M205 C). Stacks were made with the same software as the head skeletons. Drawings were elaborated from the stacks made with the same equipment.

In the ‘Material examined’ for each species, a forward slash (‘/’) separates data from different labels. The studied material is deposited in the following collections: Colección Entomológica de la Universidad de Alicante, CIBIO Research Institute (CEUA, Alicante, Spain); Museum of Comparative Zoology (MCZ, Cambridge, MA, USA; Natural History Museum (NHM, London, UK); Canadian National Collection of Insects, Arachnids and Nematodes (CNC, Ottawa, ON, Canada). The collection where each specimen or group of specimens is deposited is specified in square brackets.

### 2.3. DNA Study

The right mid leg was removed from selected adult specimens. Some legs were sent to the University of Guelph Biodiversity Institute (Guelph, ON, Canada) for sequencing of the 5’ end of the cytochrome *c* oxidase I mitochondrial gene (COI), or barcoding region, following protocols published in Hajibabaei et al. [21]. Others were processed in house at the CNC by Scott Kelso using a modified version of the same protocol, with custom primers shown in Table 1. These custom primers, COI-FX-A-R, B-F, B-R and C-F are designed to sequence the barcoding region in three portions, labelled A, B and C after the primers, increasing the chance of successfully sequencing heavily fragmented DNA. This enabled sampling of species for which only older material, considered unsuitable for barcoding, existed.

With material sequenced at CNC, raw sequence reads were scored using Sequencher 5.4.6 (2018) and aligned using Mesquite [24]. In some cases, BOLD (Barcode of Life Data System) alignments were also manually checked and corrected using Mesquite. The sequence data obtained are stored online on the BOLD database (www.boldsystems.org). Data are publicly accessible in the *Meromacrus* revision dataset, available at http://www.boldsystems.org (dx.doi.org/10.5883/DS-SYRMEROM). Sequences obtained for this project are also available on GenBank (https://www.ncbi.nlm.nih.gov/genbank/), with accession numbers listed in Table 2. Neighbour-joining (using the BOLD algorithms) was used to explore species concepts for ingroup taxa. Uncorrected pairwise genetic distances (p-distance) (see Supplementary Table S1) were calculated with Mega7 [25]. Maximum likelihood, utilizing RAxML v8 [26], was used to create a preliminary phylogenetic hypothesis. The model calculated and used in this analysis was GTR + G + I. Bootstraps were calculated using 1000 replicates. The most likely tree is presented in Supplementary Figure S1. *Quichuana calathea* Shannon, 1925 and *Tigridemyia curvigaster* (Macquart, 1842) were used as outgroups for the likelihood analysis.

## 3. Results

### 3.1. Descriptions of New Meromacrus Species

#### 3.1.1. *Meromacrus cactorum* sp. nov.

**Figure 1:**
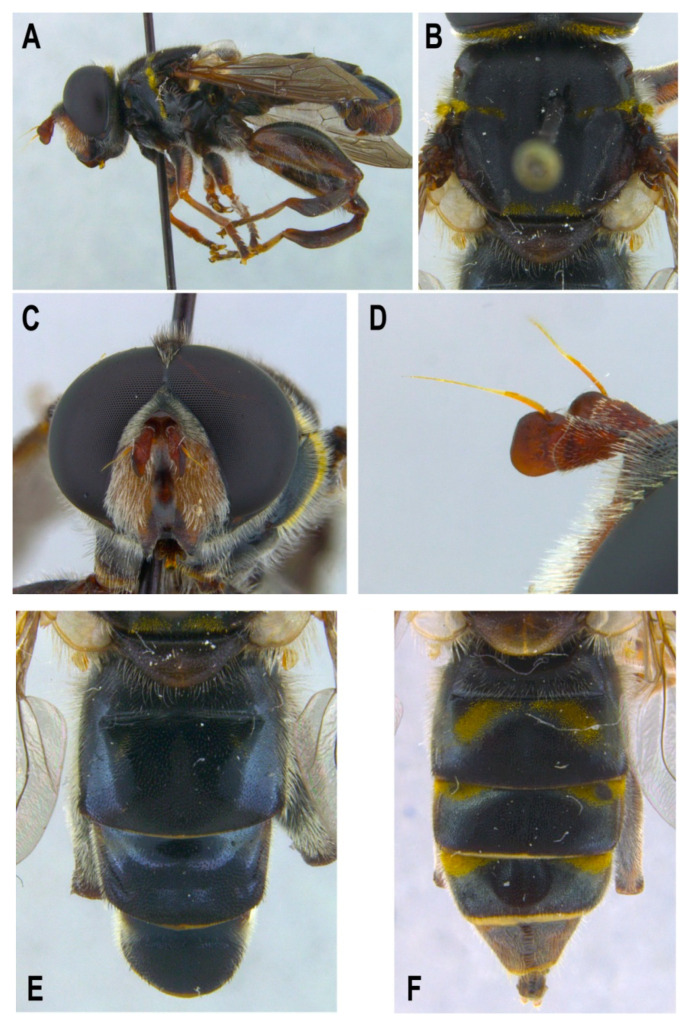
*Meromacrus cactorum* sp. nov., adult (**A**–**E**): male holotype. f: female paratype). (**A**): entire body, lateral view. (**B**): thorax, dorsal view. (**C**): head, anterior view. (**D**): antennae, lateral view. (**E**,**F**): abdomen, dorsal view.

**Figure 2:**
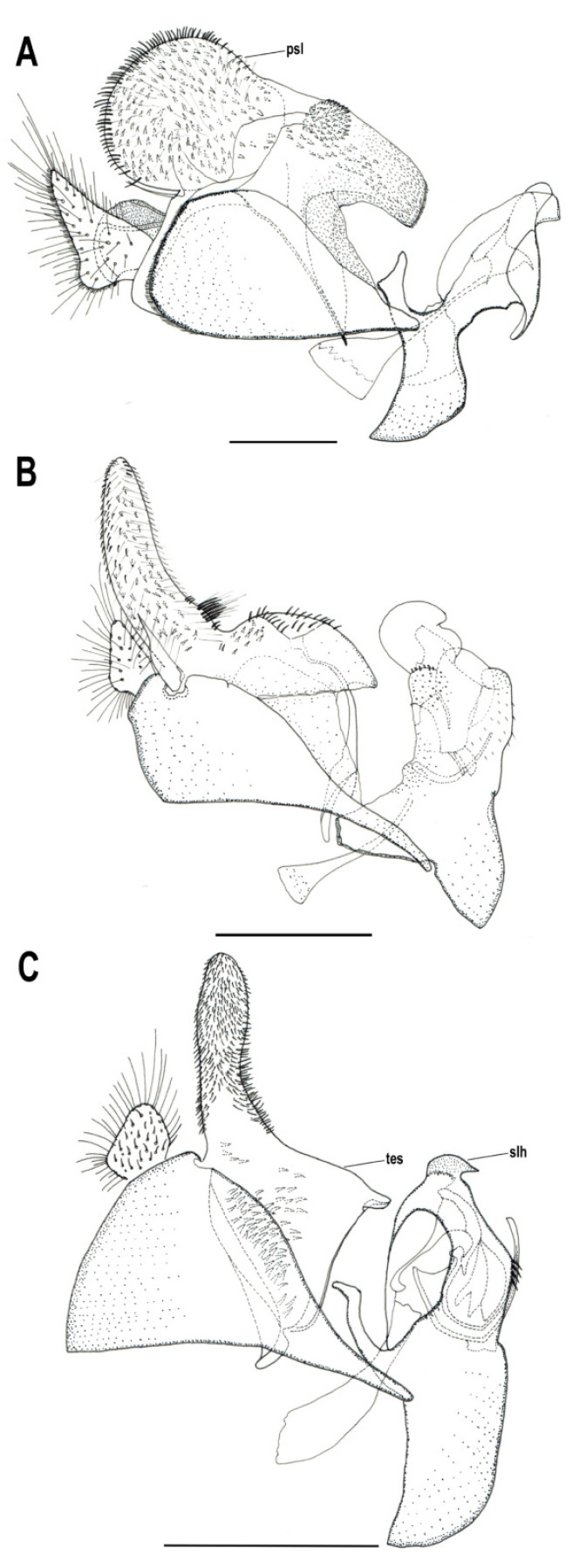
*Meromacrus* male genitalia, lateral view. (**A**): *M. cactorum* sp. nov., scale bar = 0.45 mm. (**B**): *Meromacrus ruficrus*, scale bar = 0.78 mm. (**C**): *Meromacrus yucatense* sp. nov., scale bar = 1 mm. Legend: psl, posterior surstylar lobe; tes, triangular expansion of surstylus; slh, superior lobe of hypandrium.

**Figure 3:**
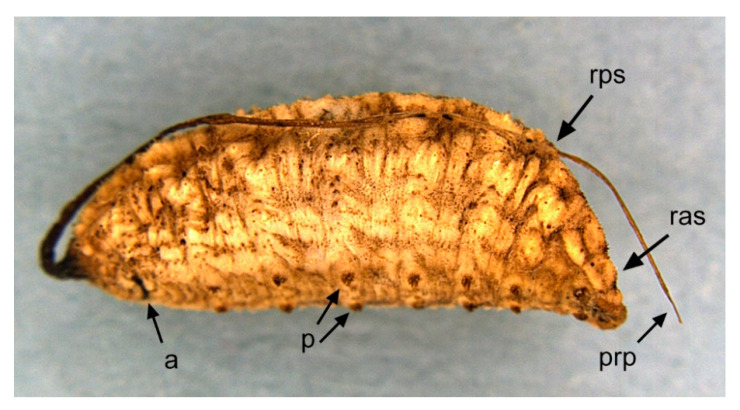
*Meromacrus cactorum* sp. nov., puparium, lateral view. Legend: a, anus; ras, region of the anterior spiracle plate; rps, region of the pupal spiracle plate; p, prolegs; prp, posterior respiratory process.

**Figure 4:**
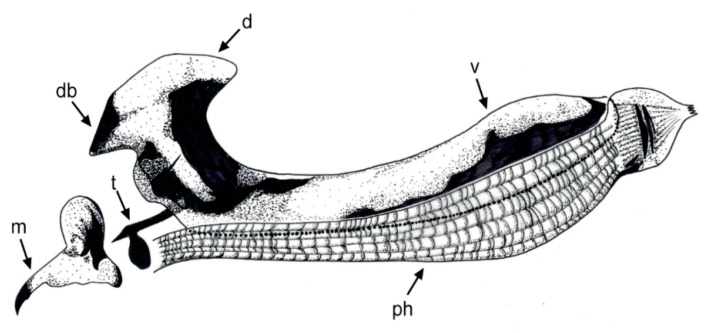
*Meromacrus cactorum* sp. nov., head skeleton, lateral view. Sclerotised areas or parts of the head skeleton are indicated in different black intensities. The pharyngeal ridges in this species are heavily sclerotised structures represented here in lighter colour than in the actual specimen. Legend: d, dorsal cornu; db, dorsal bridge; m, mandibular hook; ph, pharyngeal ridges; t, tentorial arm; v, ventral cornu. Scale bar = 250 µm.

**Figure 5:**
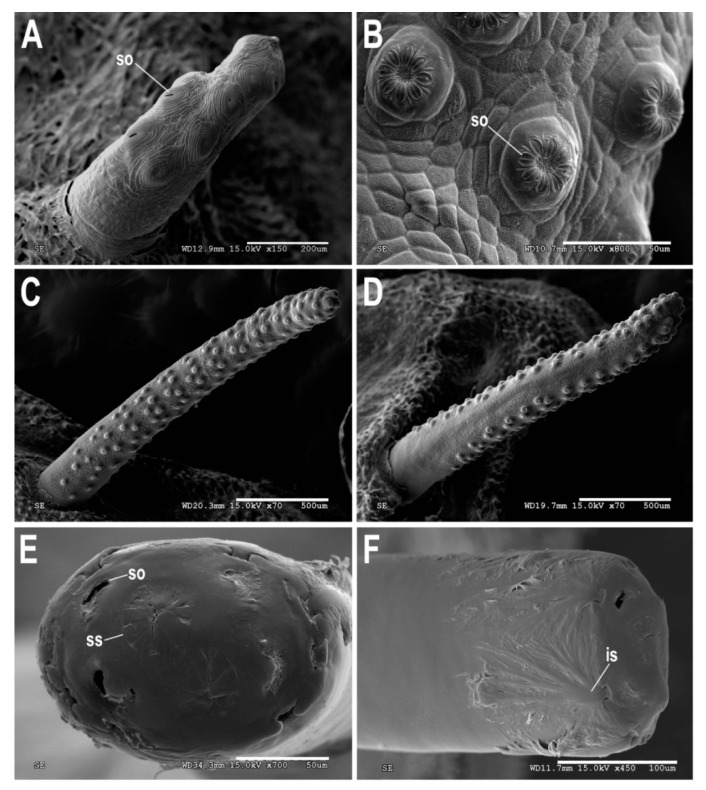
*Meromacrus cactorum* sp. nov., puparium. (**A**): anterior spiracle, ventral view. (**B**): tubercle with spiracular openings on a pupal spiracle. (**C**): pupal spiracle, dorsal view. (**D**): pupal spiracle, ventral view. (**E**): posterior respiratory process (PRP), apical view. (**F**): posterior respiratory process (PRP), dorsal view. Legend: so, spiracular opening; ss, spiracular scar; is, interspiracular setae.

Figure 1, Figure 2A, Figure 3, Figure 4 and Figure 5

**Figure 6:**
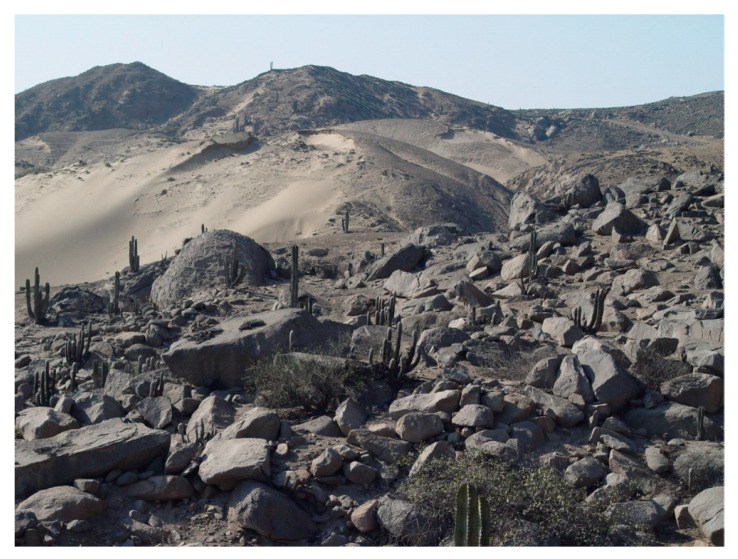
Type locality of *Meromacrus cactorum* sp. nov. (Cerro Campana, Trujillo, Peru) with *Espostoa melanostele* cacti, where larvae of this new *Meromacrus* species were found (Photo: Eduardo Galante).

- Material examined. ***Holotype***. One male with genitalia stored in a plastic microtube, reared from larva collected in decaying *E. melanostele* cacti and with preserved puparium (Perú, Trujillo, Cerro Campana), 17.01.2005, Ref. 634, Leg.: M.A. Marcos/MMM1/CEUA00006692 (bar code label) [CEUA]. ***Paratypes***. Four males and three females, all reared from larvae, with same data as the holotype: one male, Ref. 635/CEUA00006693 (bar code label); one male (genitalia not dissected), Ref. 638/MMM2/*Meromacrus* spa-2/CEUA00006689 (bar code label); one male, Ref. 633/MMM3/CEUA00006691 (bar code label) [CEUA]; one male, Ref. 636/MMM5/UA14ME (DNA analysis code)/CEUA00006690 (bar code label) [CNC]; one female, Ref. 639/CEUA00006686 (bar code label); one female, Ref. 640/*Meromacrus* spa-1/CEUA00006687 (bar code label); one female (head skeleton of puparium stored in a plastic tube), Ref. 637/UA15ME (DNA analysis code)/CEUA00006688 (bar code label) [CEUA].
- Material examined of other species. Holotype of *Meromacrus melmoth*: one male (Bolivia, Prov. Sara), Steinbach (hand written in black ink)/M.C.Z. Type, 22223 (number handwritten in black ink on a red label)/*Meromacrus melmoth* Hull (handwritten in black ink)/Ant Image Database/MCZ-ENT 00022223 (QR Code label) [MCZ]. Specimen in good condition but covered partly in fungi hyphae. Photos of the holotype available at http://mczbase.mcz.harvard.edu/name/Meromacrus%20melmoth.
- Diagnosis. This new species meets all characters and remarks stated for the genus *Meromacrus* in Blatch et al. [4], except for its wholly pilose anepimeron and virtually hyaline wing. This species can be separated from other congeneric species by the following combination of characters: general body colouration black; eyes approximated along a very short length, and separated by a distance equal to the diameter of a largest facet (only males); axe-shaped orange antenna, with pedicel longer—sometimes slightly—than basoflagellomere; face with a medial dark brown to black vitta; occiput with yellow tomentose pile on the dorsal 1/3; scutum with an inconspicuous line of yellow tomentose pile along each transverse suture and notopleuron, continued along the posterior margin of posterior anepisternum; posterior margin of scutum with two maculae of sparse yellow tomentose pile; postalar callus with sparse tomentose pile posteriorly; swollen metafemur, as broad as the width of tergum 4; metatibia curved and broad; elongate abdomen, with orange maculae at least in tergum 4; terga 2–4 with a narrow yellow fasciae on the posterior margin; male genitalia as in Figure 2A.
- Adult. MALE (holotype). Holotype size: 13 mm. Range of male sizes (n = 5): 13–14 mm. ***Head*** (Figure 1A,C). Eye with larger facets near eye contiguity; vertical triangle with dark brown to black pile, except for the short white pile on its anterior corner and the long white pile posterior to ocellar triangle; ocelli ellipsoidal, light brown; ocellar triangle slightly elevated in lateral view, and anterior corner of the vertical triangle not elevated; eyes approximated along a very short length, 4–5 facets long, and separated by a distance equal to the diameter of a largest facet; dark brown to black frontal triangle, with white pile; brown lunule; axe-shaped orange antenna, with black basoflagellomere along its dorso-apical margin; scape and pedicel with white pile; light orange arista; trapezoidal basoflagellomere, shorter than pedicel (Figure 1D); face with a medial dark brown to black vitta, elsewhere orange and sparsely pollinose, with silver white pile (Figure 1C); ventral tubercle of face slightly marked but visible; black gena, with two orange maculae—one larger than other—on each eye margin; occiput with yellow tomentose pile on the dorsal 1/3, elsewhere light *yellow* pilose anteriorly and white pilose posteriorly; occiput sparsely pollinose, black except for the narrowly orange eye margin on the dorsal 1/3. ***Thorax*.** Black scutum, black pilose except for the white pile on the anterior margin, and an inconspicuous line of yellow tomentose pile along each transverse suture and notopleuron (Figure 1B), continued along the posterior margin of posterior anepisternum; postalar callus with long white pile posteriorly intermixed with two or three tomentose pile; posterior margin of scutum with two maculae of sparse yellow tomentose pile (Figure 1B); scutum with two inconspicuous medial grey-pollinose vittae extending along the anterior 3/4 of scutum length; scutellum brown, blackish laterally, with both short black and long white pile intermixed; extensively black pleuron; posterior anepisternum, katepisternum, anepimeron and metasternum with white to light yellow pile. ***Wing***. Hyaline, extensively microtrichose, with narrow bare areas in cells R and BM basally; stigmal crossvein conspicuous; spurious vein as thick as close veins; orange pilose basicosta and black pilose tegula; calypter white centrally and light brown along the margin, with white pile; light orange halter. ***Legs***. Anterior part of all coxae white pilose; basal part of all femora with a well-defined macula of black setulae antero-ventrally; orange pro- and mesofemora, black dorsally; metafemur orange anteriorly, but black dorsally and posteriorly; white pilose pro- and mesofemora, with some black pile in mesofemur ventrally; white pilose metafemur, with thick black pile on its ventro-posterior margin basally, and its ventro-anterior margin apically (apical part with some longer black pile); swollen metafemur, as broad as the width of tergum 4 (Figure 1A); tibiae extensively orange, except for the extensively black metatibia (orange apically) (Figure 1A); all tibiae white pilose, except for a few very short setulae in the mesotibia basally and some black setae at the mesotibia apex; metatibia curved and broad, with a triangular projection posteriorly, at the apex; tarsi orange, except for the black dorsal part of tarsomeres 3–5, all tarsi white to light yellow pilose; claws black apically. ***Abdomen*** (Figure 1E). Elongate; terga black except for the orange lateral maculae in the anterior part of tergum 2, and the lateral margins of terga 3 and 4; dorsum of abdomen metallic, with greyish blue reflections; terga 2–4 with two inconspicuous maculae of white pollinosity on the anterior margin and a narrow bare yellow fascia on the posterior margin; all terga black pilose, except for the white to light yellow pile on antero-lateral areas of each tergum and lateral margins; pleural membranes orange; sterna extensively orange, with long orange pile. ***Genitalia***. Posterior surstylar lobe broad and roundish, black pilose (Figure 2A). FEMALE. Range of female sizes (n = 13.5–13.75 mm). Similar to male except for the following characters: frons with a fascia of sparse white pollinosity; frons orange and white pilose on the ventral 3/4; basoflagellomere nearly as long as pedicel; grey pollinose vittae of scutum even less conspicuous than in male; posterior part of postalar callus with yellow tomentose pile connecting with a tomentose fascia on posterior margin of scutum; cells R and BM with bare areas basally; basal part of metafemur without black pile; metatibia without a triangular projection posteriorly, at the apex; terga 2–4 with two maculae of tomentose yellow pile on the anterior margin, united in tergum 2; at least tergum 4 with some orange parts (Figure 1F).
- Taxonomic notes. *M. cactorum* sp. nov. does not key out using Hull (1942) due to its hyaline loop of vein R4 + 5 and the two medial grey-pollinose vittae on scutum. However, this species appears to belong to the group of ‘very dark, black or almost black flies’ referred to in the couplet 1 of the key in Hull [20]. Within this group, *M. cactorum* sp. nov. can be readily separated from *Meromacrus melmoth* Hull, 1937 and *Meromacrus pluto* Hull, 1942 by the shape of basoflagellomere, which is about as long as wide, blunt apically in *M. cactorum* sp. nov. (Figure 1D), while elongate, slightly concave dorsally and curved at its pointed apex in *M. melmoth* and *M. pluto* [Hull [20]: Figure 13]. In addition, dorsum of abdomen has greyish blue metallic reflections in *M. cactorum* sp. nov. male, while in *M. melmoth* (holotype) is dull. The dark species *Meromacrus niger* Sack, 1920 [= *Meromacrus funereus* Shannon and Aubertin, 1933, according to Pape & Thompson [27]] has the metafemora less thickened than *M. cactorum* sp. nov. and the basoflagellomere broadly rounded, not trapezoidal as in the new species (Figure 1D). *M. cactorum* sp. nov. is also similar to *Meromacrus brunneus* Hull, 1942 due to the general shape of antenna and very thickened metafemur, but *M. cactorum* sp. nov. has the wing extensively hyaline, and tomentose pile on transverse suture, notopleuron (Figure 1B) and posterior anepisternum, while *M. brunneus* has the anterior margin of wing brown pigmented, a line of tomentum between postpronotum and transverse suture and pleuron without tomentum.
- Etymology. The specific epithet ‘cactorum’ refers to the cacti, which are the breeding sites of this species.
- Puparium. ***Shape and size*** (Figure 3). Subcylindrical, tapered posteriorly, with a typical eristaline long tail. Light brown. Tegument slightly punctured with spicules. 6 pairs of prolegs on small cones, with numerous crochets. 11.82 mm long (10.66–12.46), 4.70 mm high (4.42–4.89) and 5.88 mm wide (5.56–6.17) (n = 4). ***Head skeleton*** (Figure 4). Heavily sclerotised, especially on the anterior and posterior margins of the dorsal cornu and the rear part of the ventral cornu. Dorsal cornu shorter than ventral cornu. In profile view, dorsal bridge area in acute angle. Mandible with hooks present but not much developed, sclerotised at their tips. ***Anterior spiracles*** (Figure 5A). Straight structures, light brown and shiny, almost 3 × longer than broad at the base, with paired linear-shaped openings all along the ventral surface of the tube. Smooth and reticulated surface, ridges concentrically arranged around the openings. ***Pupal spiracles*** (Figure 5B–D). Subcylindrical and slightly curved tubes, dark brown and less shiny than the anterior spiracles, ≈1.9 mm long, more than 6 × longer than broad at the base. Straight, slightly curved at the tip. Surface reticulated, with 14–16 bands of spiracles arranged almost at the base of the tube, absent on the ventral surface. Each band with 8–12 tubercles, each one bearing 5–8 oval spiracular openings. ***PRP*** (Figure 5E,F). Subcylindrical to oval in cross section, ≈167 µm broad near the apical end of the structure. Surface clear and smooth, without any apparent transverse ridge (maybe hidden by the tegument). Spiracular plate domed, with two twisted central scars, two pairs of curved openings and four pairs of feathery interspiracular setae, highly divided and covering the distal perimeter of the PRP.
- Biology and habitat. Larvae were collected in the *E. melanostele* cacti of an extremely arid area from Peru where cacti dominated the vegetation (Figure 6).
- Larvae were collected in cactus cavities containing wet decaying tissues, particularly in fallen or dead parts of cacti. Larvae of *M. cactorum* sp. nov. coexisted in the same breeding site with at least two species of *Copestylum* Macquart, 1846, *Copestylum cockerelli* (Curran, 1927) and *Copestylum hambletoni* (Fluke, 1951) [28].

#### 3.1.2. *Meromacrus yucatense* sp. nov.

**Figure 7:**
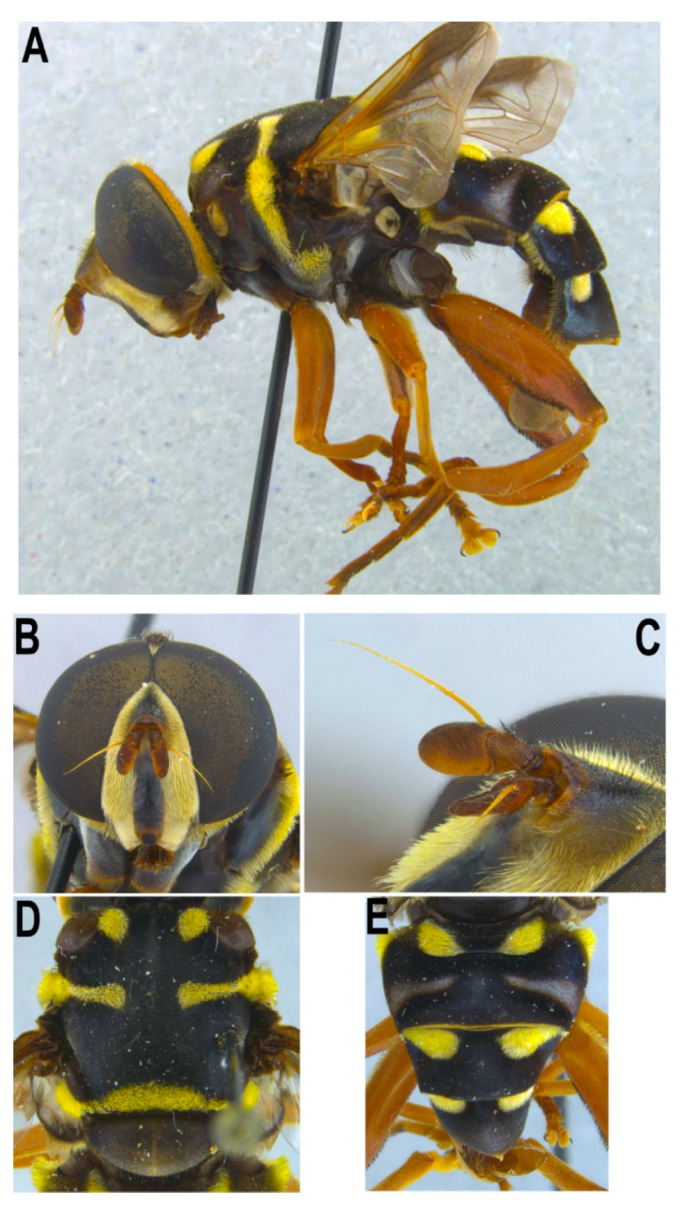
*Meromacrus yucatense* sp. nov., adult, male holotype. (**A**): entire body, lateral view. (**B**): head, anterior view. (**C**): right antenna, lateral view of inner side. (**D**): thorax, dorsal view. (**E**): abdomen, dorsal-lateral view.

**Figure 8:**
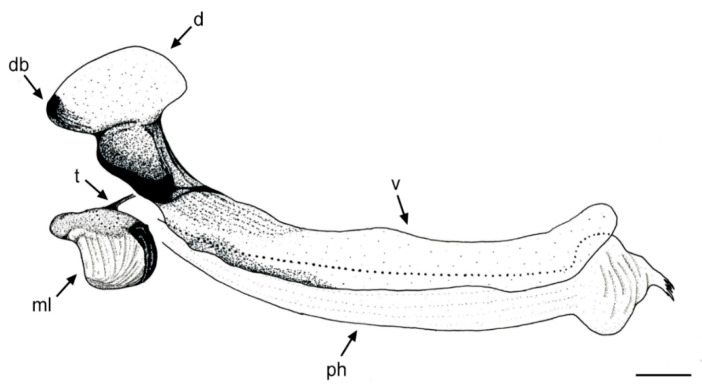
*Meromacrus yucatense* sp. nov., head skeleton, lateral view. Sclerotised areas or parts of the head skeleton are indicated in different black intensities. The pharyngeal ridges in this species are poorly sclerotised and inconspicuous structures. Legend: d, dorsal cornu; db, dorsal bridge; ml, mandibular lobe; ph, pharyngeal ridges; t, tentorial arm; v, ventral cornu. Scale bar = 250 µm.

**Figure 9:**
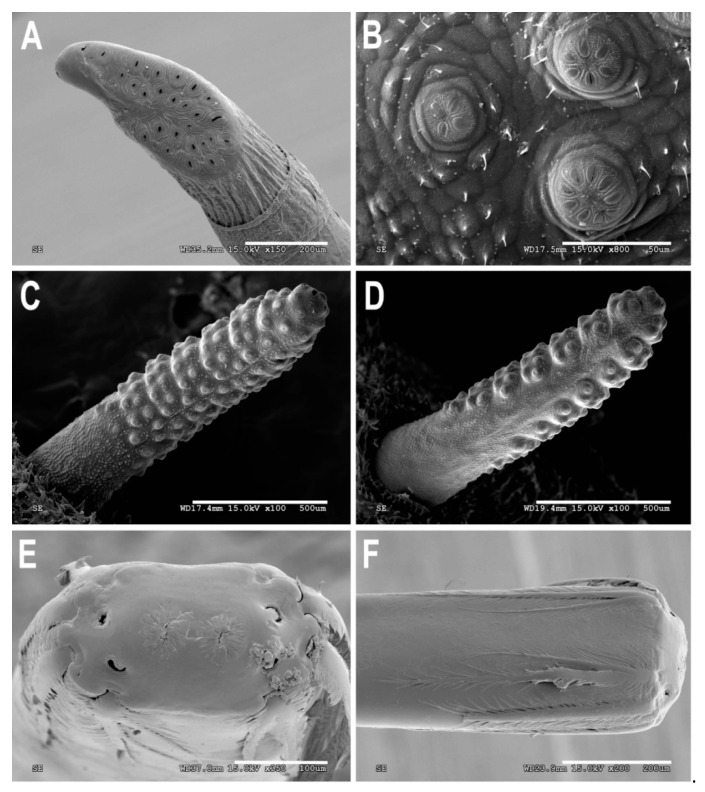
*Meromacrus yucatense* sp. nov., puparium. (**A**): anterior spiracle, ventral view. (**B**): tubercle with spiracular openings on a pupal spiracle. (**C**): pupal spiracle, dorsal view. (**D**): pupal spiracle, ventral view. (**E**): posterior respiratory process (PRP), apical view. (**F**): posterior respiratory process (PRP), dorsal view.

Figure 2C, Figure 7, Figure 8 and Figure 9

**Figure 10:**
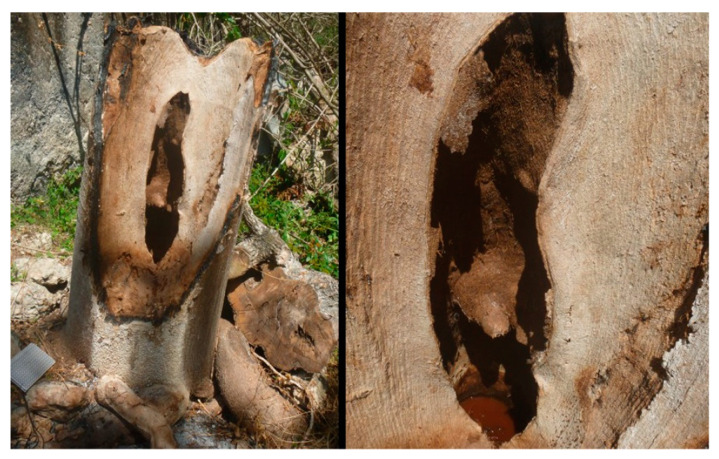
Stump of *Ceiba pentandra* (**left**) showing the water-filled rot hole (**left** and **right**) where larvae of *Meromacrus yucatense* sp. nov. were found (Photo: Javier Quinto).

- Material examined. ***Holotype***. one male with genitalia stored in a plastic microtube, reared from larva and with preserved puparium: (Yabucú (Acanceh), Yucatán, México), 20.81192, -89.41275, 15.03.2014, en *C. pentandra* (Malvaceae), Leg.: J. Quinto/SYRPHIDAE Meromacron [misspelling of *Meromacrus*] sp 44 [specimen 44], oquedad en tronco podado [‘hole in pruned trunk’], L 15-3-14, P 2-4-14, A 14-4-14, Det. J. Quinto 2014/MMY1 [hand written]/7 [hand written] [CEUA]. ***Paratypes***. Three males with genitalia stored in a plastic microtube, with preserved puparia: same locality data as the holotype, all identified as SYRPHIDAE Meromacron [misspelling of *Meromacrus*] by J. Quinto 2014, and reared from larvae collected in ‘oquedad en tronco podado’ [hole in pruned trunk]/sp 28, L 15.3.14, P 27.3.14, A 8.4.14/MMY2 [hand written]/UA13ME [hand written, DNA analysis code] [CEUA]; sp 38, L 15.3.14, P 27.3.14, A 9.4.14/MMY3 [hand written]/UA12ME [hand written, DNA analysis code]/8 [hand written] [CNC]; sp 9, L 22.3.14, P 3.4.14, A 9.4.14/6 [hand written] [CEUA].
- Diagnosis. This new species meets all characters and remarks stated for the genus *Meromacrus* in Blatch et al. [4] and it can be separated from other congeneric species by the following combination of characters: antenna orange; basoflagellomere oval, over 1.3 times longer than width (holotype) (Figure 7C); face with a medial black vitta; scutum with a tear-shaped macula of golden-yellow tomentose pile on the anterior margin, next to each postpronotum, a line of golden-yellow tomentose pile along each transverse suture and notopleuron, continued along the posterior margin of posterior anepisternum and dorsal margin of katepisternum; postalar callus with a tuft of golden-yellow tomentose pile connecting with a semicircular fascia of tomentose pile along the entire posterior margin of scutum; legs extensively orange, with a black carina on the basal 1/3 of metatibiae ventrally; tergum 2 with two lateral slender triangular whitish-yellow markings; tergum 1 with two triangular maculae of golden yellow tomentose pile; terga 3 and 4 with two oval maculae of tomentose pile on the anterior margin of each terga; male genitalia as in Figure 2C.
- Adult. MALE (holotype). Holotype size: 17.25 mm. Range of male sizes (n = 4): 14.5–17.25 mm. ***Head*** (Figure 7A,B). Eye with larger facets near eye contiguity; ocellar triangle slightly elevated in lateral view, with dark brown to black pile progressively longer towards the occiput; ocelli ellipsoidal, light brown; anterior corner of the vertical triangle not elevated in lateral view, slightly white pollinose and with short silver-white pile; eye contiguity 16–17 facets long; dark brown frontal triangle, with black pile, white pollinose and with silver white pile laterally; light brown lunule; orange antenna, slightly darkened in the dorsal part of basoflagellomere (Figure 7C); scape and pedicel with black setulae of different lengths; light orange arista; oval basoflagellomere, about 1.3 times longer than wide (Figure 7C); face with a medial black vitta (Figure 7B), elsewhere white pollinose, with silver white pile; ventral tubercle of face inconspicuous, nearly absent; gena light orange with darker areas; occiput with light-orange tomentose pile, except the area just behind the vertical triangle. ***Thorax***. Scutum black, with brown postpronotum; scutum with a tear-shaped macula of golden-yellow tomentose pile on the anterior margin, next to each postpronotum; scutum with a line of golden-yellow tomentose pile along each transverse suture and notopleuron (inner end of line widened), continued along the posterior margin of posterior anepisternum and dorsal margin of katepisternum (Figure 7D); postalar callus with a tuft of golden-yellow tomentose pile connecting with a semicircular fascia of pile of the same kind along the entire posterior margin of scutum; scutum with a medial grey-pollinose vitta extending along the anterior 3/4 of scutum length, a fainter grey-pollinose vitta from each tear-shaped tomentose macula to the transverse suture, and an equally faint pollinose macula next to each postalar callus; scutellum brown, darker on the anterior margin, with short black pile all over, except for a line of light brown pile on its posterior margin; posterior anepisternum with golden regular yellow pile, next to the tomentose line; katepisternum with regular yellow pile, longer ventrally; anepimeron with fine yellow pile, and black pile postero-dorsally; metasternum black pilose. ***Wing***. Wholly microtrichose, brown pigmented on the anterior margin, except cell C; brown pigmentation darker apically than basally, and not extending beyond the apical end of cell R2 + 3; stigmal crossvein conspicuous; spurious vein as thick and sclerotised as close veins; orange pilose basicosta and black pilose tegula; calypter white centrally and black along the margin, with light brown pile; white halter. ***Legs***. Extensively orange (Figure 7A), with a black carina on the basal 1/3 of metatibiae ventrally; anterior part of all coxae with both black and orange pile intermixed; basal part of all femora with a well-defined macula of black setulae antero-ventrally, more anterior than ventral in metafemora; all femora with black pile ventrally, and a bare line apico-ventrally; metafemur with setulae apico-ventrally; dorsal part of all femora with black pile, specially abundant in metafemora apically; tibiae extensively orange pilose, with scattered short black pile; all tarsomeres with at least one or two black pile dorsally, usually extensively black pilose; all tarsi orange pilose ventrally; claws black apically. ***Abdomen***. Terga black except for two lateral slender triangular whitish-yellow markings on tergum 2 (Figure 7E); all terga with short black pile, except the following parts: tergum 1 with two triangular maculae of golden yellow tomentose pile; terga 3 and 4 with two oval maculae of tomentose pile on the anterior margin of each terga, each macula nearly reaching the midpoint of tergum; regular yellow pile present on the anterior corner of tergum 2 and along the lateral margins of terga 2-4; pleural membranes and sterna black; all sterna with long yellow pile, except for the black pile of sternum 4. ***Genitalia***. Posterior surstylar lobe elongated, straight apically, slightly expanded before the round apex; basal part of surstylus with a triangular expansion that curves inwards; surstylus black pilose all over, with a patch of thicker setae on the inner part; superior lobes of hypandrium anteriorly curved, pointed at apex (Figure 2C). FEMALE. Unknown.
- Taxonomic notes. *M. yucatense* sp. nov. does not key out using the key of Mesoamerican *Meromacrus* in Blatch et al. [4] due to the black facial vitta, light brown basoflagellomere, and orange pilose basicosta all in combination. This species and *Meromacrus currani* Hull, 1942 have a similar thoracic pattern of tomentose pile ([4]: Figure 3), but they can be separated by the shape of the yellow triangular markings of tergum 2, which in *M. yucatense* sp. nov. are tapering towards their inner ends Figure 7E), as in *M. laconicus*, and in *M. currani* are widening ([4]: Figures 5 and 6). The male genitalia of *M. yucatense* sp. nov. and *M. currani* are also very different, with a round cerci and a straight surstylus apex in *M. yucatense* sp. nov. (Figure 2C), and triangular cerci and a recurved surstylus apex in *M. currani* ([4]: Figure 8A–C). *M. yucatense* sp. nov. can be distinguished from the similar *M. laconicus* in the shape of the tomentose maculae of terga 3 and 4, which are oval in *M. yucatense* sp. nov. (Figure 7E) and linear in *M. laconicus* ([4]: Figure 6). In addition, these two species differ in the shape of the surstylus, as shown in Figures 2C and 10A of Blatch et al. [4].
- Etymology. The specific epithet ‘yucatense’ refers to the state of Yucatan (Mexico), where the type locality of this species is found.
- Puparium. ***Shape and size***. Subcylindrical, tapered posteriorly, with a typical eristaline long tail. Brown in colour. Tegument slightly punctured with spicules. 6 pairs of prolegs on small cones, with numerous crochets. 10.9 mm long (10.71–11.2), 5.49 mm high (5.44–5.53) and 6.47 wide (6.24–6.64) (n = 3). ***Head skeleton*** (Figure 8). In general, of the filter-feeding type [19], heavily sclerotised only in the area between the dorsal bridge and the tentorial arm. Dorsal cornu shorter than ventral cornu. In profile view, dorsal bridge area in obtuse angle. Mandible without hooks. ***Anterior spiracles*** (Figure 9A). Straight structures, light brown and shiny, striated surface along the tube, 3× longer than broad at the base, slightly curved at the end. Numerous respiratory openings on a plate at the ventral tip of the tube. Surface of the plate reticulated and smother than the rest of the entire structure, ridges concentrically arranged around the spiracular openings. ***Pupal spiracles*** (Figure 9B–D). Subcylindrical and slightly curved tubes, dark brown, ≈1.2 mm long, more than 3.5 × longer than broad at the base. Surface finely granulated or reticulated, smoother to the apex. 7–8 apparent bands of spiracular tubercles arranged along the 3/4 upper part of the tube, absent on the ventral area. Each band with 10–18 respiratory tubercles, with 4–9 spiracular oval-shaped openings. Surface bearing spiracles with both straight and curved setae between the tubercles. ***PRP*** (Figure 9E–F). Almost rectangular in cross section, dorso-ventrally flattened, ≈300 µm broad near the apical end of the tube. Surface clear and smooth, without any apparent transverse ridge. Spiracular plate with two central scars, two pairs of curved openings and four pairs of feathery interspiracular setae, dorsal and ventral pairs bifid, one branch bigger than the other; lateral pairs not bifid, robust and uniramous.
- Biology and habitat. Larvae were found in a traditional henequen (*Agave fourcroydes* Lem., Asparagaceae) hacienda. By the 1850s, the henequen industry collapsed and natural vegetation colonized large areas cultivated with henequen. The studied hacienda is now embedded in a heterogeneous landscape matrix, including remnants of tropical secondary dry forest with large old trees, and agriculture and livestock areas in which crop rotations and different types of management take place.
- Larvae were collected in a single *Ceiba pentandra* stump with a large water-filled tree hole containing abundant wood decay (Figure 10). This stump was the result of a recent pruning at ground level of an old tree (the margins were burned to prevent regrowth), exposing the hole that the trunk had inside. Larvae of three *Meromacrus* species, *M. gloriosus*, *M. laconicus* and *M. yucatense* sp. nov., were found coexisting in the same hole. All the larvae of *M. yucatense* sp. nov. pupated between 22 March and 3 April, they stayed as pupae during 13–14 days and adults emerged between 3 and 14 April.

### 3.2. Identification Key to Meromacrus puparia (Based on Pérez-Bañón et al. [9])

(1) Pupal spiracles with the tubercle bands reaching the ventral surface...............................................2
  Pupal spiracles with the tubercle bands do not reaching the ventral surface....................................4
(2) Tubercle bands reach the base of the pupal spiracle on the dorsal surface. Bands clearly separated on the dorsal surface even on the basal part..........................................................*Meromacrus currani*
  Tubercle bands do not reach the base of the pupal spiracle.....................................................................3
(3) Anterior spiracles two times longer than broad. Pupal spiracles with the tubercles only arranged in bands at the edges of the spiracles..........................................................................*Meromacrus draco*
  Anterior spiracles three times longer than broad. Pupal spiracles with the tubercles arranged in bands not only at the edges of the spiracles, but also on the dorsal surface; the bands are not clear on the basal part.............................................................................................................*Meromacrus laconicus*
(4) Pupal spiracles clearly tapering apically....................................................................................................5
  Pupal spiracles only slightly tapering apically...........................................................................................6
(5) Pupal spiracles with over 75% of their dorsal and lateral surfaces covered with 6–8 bands of tubercles. Anterior spiracles with spiracular openings arranged on a ventral and flat plate...................................................................................................................................*Meromacrus acutus*
  Pupal spiracles with almost their entire lateral and dorsal surfaces covered with 14–16 bands of tubercles (Figure 5C). Anterior spiracles with spiracular openings arranged in pairs along their ventral curved surfaces (Figure 5A)........................................................*Meromacrus cactorum* sp. nov.
(6) Anterior larval spiracles two times longer than broad............................................*Meromacrus loewi*
  Anterior larval spiracles three times longer than broad..........................................................................7
(7) Ventral surface of pupal spiracles without ridges (Figure 9D). Band area of the pupal spiracles with scarce setae (Figure 9C). PRP dorso-ventrally flattened, with two morphotypes of interspiracular setae..............................................................................................................*Meromacrus yucatense* sp. nov.
  Ventral surface of pupal spiracles with ridges. Pupal spiracles without setae on the surface........8
(8) Anterior larval spiracles slightly swollen apically. Ventral surface of pupal spiracles furrowed for many deep longitudinal carinae...............................................................................*Meromacrus obscurus*
  Anterior larval spiracles not swollen apically. Ventral surface of pupal spiracles smooth or with very superficial longitudinal ridges......................................................................*Meromacrus laconicus*

### 3.3. A New Synonymy in the Genus Meromacrus

#### *Meromacrus gloriosus* Hull, 1941

*Meromacrus draco* Hull, 1942 syn. nov. 

Figure 11

**Figure 11:**
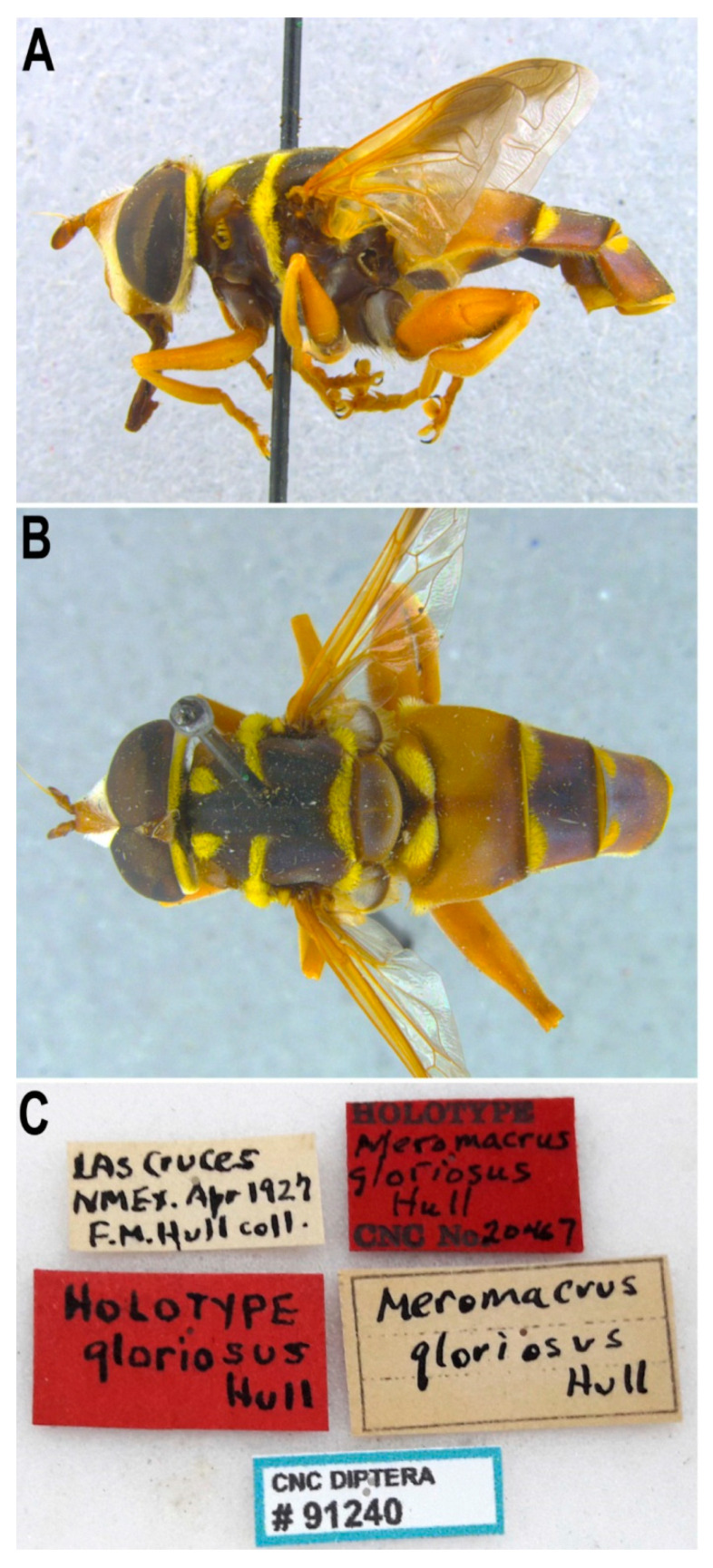
*Meromacrus gloriosus*, adult, male holotype. (**A**): entire body, lateral view. (**B**): entire body, dorsal view. (**C**): specimen labels.

- Material examined. ***Costa Rica***: Two females with puparia attached to the pin (CEUA00089990, 00089991) (Guatuso, Finca Blanco), 24.6.2009 ex larva, tallo de banano (‘stem of banana tree’), leg. M.A. Marcos García; ***Mexico***: Seven males and seven females (all except for one male and three females with puparia attached to the pin), (Yabucú (Acanceh), Yucatán, México), 20.81192, -89.41275, 15.03.2014, en *Ceiba pentandra*, oquedad en tronco podado (‘hole in a pollard’), leg.: J. Quinto, L 15.3.14, P: 25.3.14, A: 6.4.14, det. as *Meromacron* sp 16 by J. Quinto (1 female), L 15.3.14, P: 28.3.14, 9.4.14, det. as *Meromacron* spm 36 by J. Quinto (1 male), L 15.3.14, P: 28.3.14, 8.4.14, det. as *Meromacron* spm 30 by J. Quinto 2014 (1 male), L 15.3.14, P 21.3.14, A 2.4.14, det. as *Meromacron* spm 10 by J. Quinto 2014 (1 male), L 15.3.14, P 19.3.14, A 2.4.14, det. as *Meromacron* spm 5 and 6, by J. Quinto 2014 (2 males), L 15.3.14, P 20.3.14, A 2.4.14, det. as *Meromacron* spm 7 by J. Quinto 2014 (1 male), L 15.3.14, P 27.3.14, A 7.4.14, det. as *Meromacron* spm 14 by J. Quinto 2014 (1 female), L 15.3.14, P 26.3.14, A 26.4.14, det. as *Meromacron* spm 2 and 3 by J. Quinto 2014 (1 male and 1 female), L 15.3.14, P 27.3.14, A 9.4.14, det. as *Meromacron* spm 37 by J. Quinto 2014 (1 female), L 15.3.14, P 31.3.14, A 9.4.14, det. as *Meromacron* spm 34 by J. Quinto 2014 (1 female), P 15.3.2014, A 25.3.14, det. as *Meromacron* spm 37 by J. Quinto 2014 (1 female); 1 male and 1 female, huerta Cozalapa, Cd. Hidalgo, Chis., 9:30am (male), 11:05 (female), S/mango, 15.2.1990, Eslava, leg.; 1 male and 1 female, Chiapas, Ciudad Hidalgo, 21.XI.91, M.A. Ciparroa Ex mango; ***USA***: Holotype of *M. gloriosus* (Figure 11): 1 male, Las Cruces NMEx. Apr 1927 F.M. Hull coll. (hand written) / HOLOTYPE *Meromacrus gloriosus* Hull CNC No 20467 (red label) / HOLOTYPE *gloriosus* Hull (red label) / *Meromacrus gloriosus* Hull / CNC DIPTERA # 91240. Genitalia dissected and stored in a plastic microvial attached to the pin. Additional material: 1 female, AZ Santa Cruz Co. Sycamore Cn 1200m, 31º25′N 111º10′W 19.IX.01 G & M. Wood, CNC DIPTERA # 106257, Barcode of Life, DNA voucher specimen, Sample ID: C. DIPTERA 106257, BOLD Proc. ID: CNCDB3550-11, *M. gloriosus* det Skevington?; 1 female, at flowers of *Baccharis glutinosa* Pers., (Limpia Canyon, 5000ft, Davis Mta. Jeff Davis Co., Texas, USA), July 22 1946 H. E. Evans, Frank M. Hull Collection, C.N.C. 1973, CNC DIPTERA # 231463, *M. gloriosus* det Vockeroth? [CNC];
- Taxonomic notes. All examined specimens from Costa Rica (Guatuso) and Mexico (Chiapas, Hidalgo and Yucatan) are in accordance with the description of *M. draco* provided by Blatch et al. [4], who also examined the male holotype of *M. draco* at the American Museum of Natural History. In our specimens, the female frons is brown to black on the posterior half to two thirds; tegula with black pile anteriorly (at least one or two); basicosta orange pilose; metafemur black centrally along a variable length (usually narrowly orange basally and on the apical fourth) and black pilose except for the yellow pile dorsally on baso-anterior half; tergum 2 with two lateral orange maculae of variable extension, with a T-shaped black macula on the anterior margin or a H-shaped black maculae extending from the anterior to the posterior margin; terga 3–5 from black to reddish black; tergum 3 with two maculae of tomentose pile on the anterior margin; tergum 4 with two smaller tomentose maculae on the anterior margin, usually inconspicuous, sometimes virtually absent; terga 3 and 4 extensively short black pilose; sterna black to brownish black. The examined males shared the same genitalia (see Figure 9A–C in Blatch et al. [4]).

The male holotype of *Meromacrus gloriosus* (Figure 11), from the USA (New Mexico), is in general lighter than the Costa Rican and Mexican specimens, and differs from them in the following characters: tegula wholly yellow pilose; metafemur wholly orange and yellow pilose, just with black setulose pile ventrally; tergum 2 almost wholly orange (Figure 11A); terga 3 and 4 with more abundant short yellow pile, extending towards the central parts of terga; sternum 1 yellow posteriorly; sternum 2 yellow, with a central black macula. However, the holotype has the same genitalia morphology as the specimens examined from Costa Rica and Mexico. We also examined two females from the USA (Texas and Arizona, respectively). According to the key in Hull [20] these two females would not key out further than couplet 26. The female from Texas had the metafemur wholly orange but the short black pile were more abundant on its apico-posterior third than in the holotype. In addition, this female had black pile on the tegula and a T-shaped black macula on the anterior margin of tergum 2 (as *M. draco*), but yellow pile on terga 3 and 4 were nearly as abundant as in the holotype of *M. gloriosus*. The other female from Arizona was similar to the male holotype of *M. gloriosus* in having the tegula wholly yellow pilose and the metafemur extensively yellow pilose dorsally, anteriorly and posteriorly. However, the metafemur was black centrally and the tergum 2 had a T-shaped black macula on the anterior margin, as in *M. draco*. The holotype of *M. gloriosus* and both examined females had conspicuous yellow tomentose maculae on the anterior margin of terga 3 and 4, consistent with a specimen of *M. draco* from Hidalgo, Mexico. 

All this variation in the stated characteristics (otherwise, all examined specimens of *M. draco* and *M. gloriosus* were similar) seem to support the existence of a single variable taxon, as shown by the COI study of two *gloriosus*-like specimens from USA (106257: female; 106256), two *draco*-like specimens (UA1ME: male; UA2ME: female) from Yucatan, Mexico and a female from Costa Rica (UA5ME) plus six specimens from Costa Rica identified as *M. draco* in BOLD systems; all 11 specimens analysed had very similar COI sequences (see Section 3.4.4). On the basis of this evidence, both morphological and molecular, we propose *M. draco* as junior synonym of *M. gloriosus*.

### 3.4. Additional Results for Other Meromacrus Species

#### 3.4.1. *Meromacrus canusium* (Walker, 1849)

Figure 12

**Figure 12:**
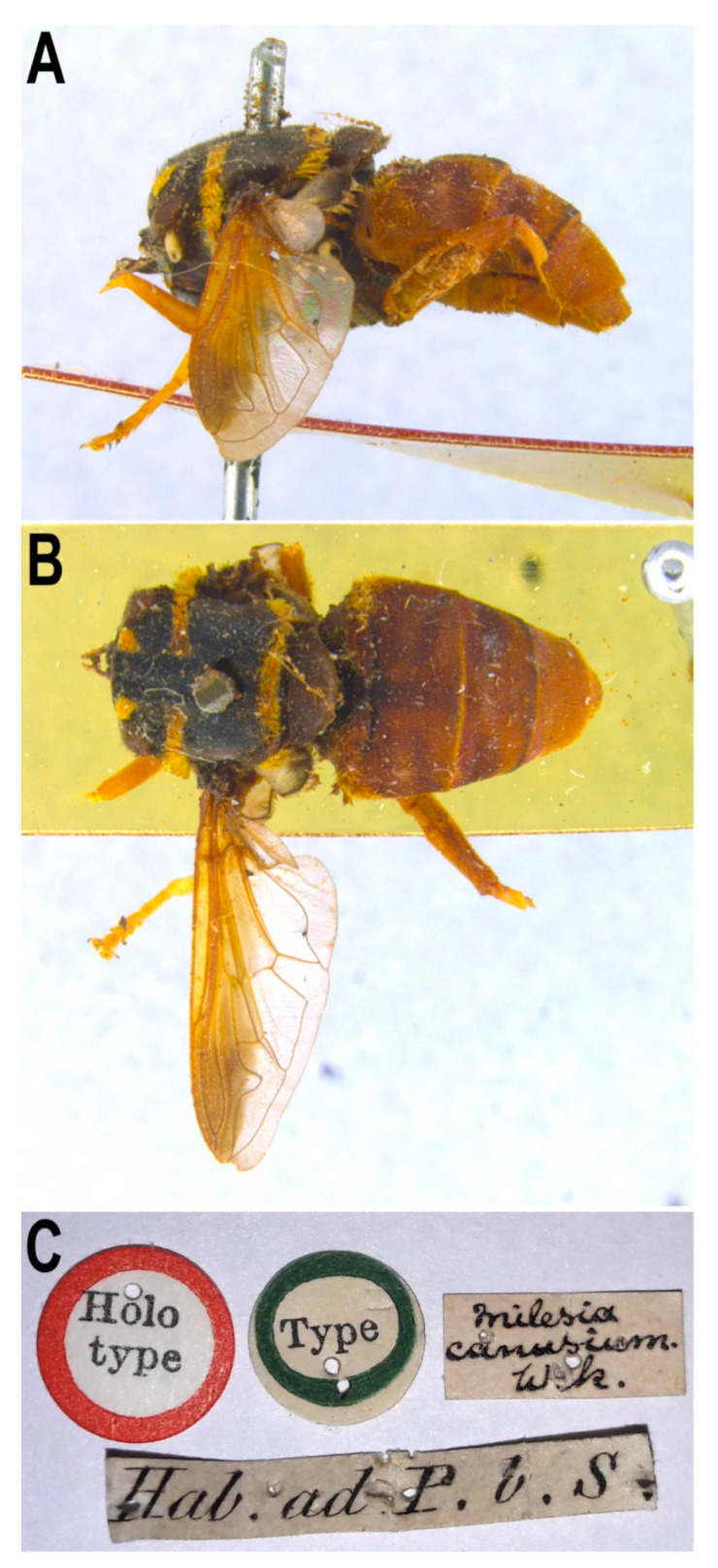
*Meromacrus canusium*, adult, female holotype. (**A**): entire body, lateral view. (**B**): entire body, dorsal view. (**C**): specimen labels.

- Material examined. Holotype (Figure 12): 1 female, Holotype (printed in a circular label with red margin)/Type (printed in a circular label with blue/green margin)/*Milesia canusium*. Wlk. (hand written)/Hab. Ad P. b. S. [NHM]. Specimen in poor condition, apparently disturbed by a liquid, headless, without right wing, left pro-and metatibiae, left pro-and metatarsus, and right legs except for the mesofemur; meso-and metafemora partly eaten by *Anthrenus*.
- Taxonomic notes. This species was described from a female of ‘Cape of Good Hope’ (Africa) under the genus *Milesia* (Walker, 1849). In his revision of the genus *Meromacrus*, Hull [20] redescribed the holotype and addressed the supposed type locality error on the basis of the exclusively Neotropical distribution of the known species of this genus. Blatch et al. [4] also examined the holotype of *M. canusium* and redescribed the species, stating that the tegula is orange pilose, metafemur extensively black, terga 2–4 with black vittae, terga 3–4 with a small yellow tomentose fasciate macula on the anterior margin, tergum 4 black pilose apico-medially and all sterna brown coloured. Neither Walker [29] nor Hull [4] addressed the tomentose macula on the anterior margin of terga 3–4. We examined the holotype and, even though the specimen is in poor condition, a close examination of it reveals no maculae of tomentose pile on these terga (Figure 12A,B); in addition, the tegula is black pilose, metafemur, terga 2–4, tergum 4 pile and all sterna wholly orange. We did not find specimens with the same combination of characters as the holotype of *M. canusium* and possibly neither did Blatch et al. [4], who apparently considered the differences with the holotype as to be intraspecific variability. This species is most similar to *M. draco* sensu Blatch et al. [4], from which these authors distinguished it by the mainly orange abdomen (mainly dark brown to black in *M. draco*) and in the female, the wholly orange frons (brown in dorsal 2/3 in *M. draco*). Apart from the holotype, Blatch et al. [4] only found a male and two females of the putative *M. canusium*, while 19 males and 21 females fit their *M. draco* concept.

The holotype of *M. canusium* is also similar to that of *M. gloriosus*. However, the holotype of *M. canusium* has the tegula wholly black pilose, terga 2–5 (Figure 12A,B) and all sterna wholly orange and tergum 4 wholly yellow pilose, while in *M. gloriosus* the tegula is yellow pilose (at most with sparse black pile anteriorly), terga and sterna are partly black or reddish black and tergum 4 with extensive areas covered in black pile. It might be that the holotype of *M. canusium* is an extreme variant of *M. gloriosus* but given the uncertain origin of the *M. canusium* holotype and its apparently unique combination of characters, we maintain this species as valid until morphological and molecular analyses of new holotype-like specimens are undertaken.

#### 3.4.2. *Meromacrus laconicus* (Walker, 1852)

Figure 13

**Figure 13:**
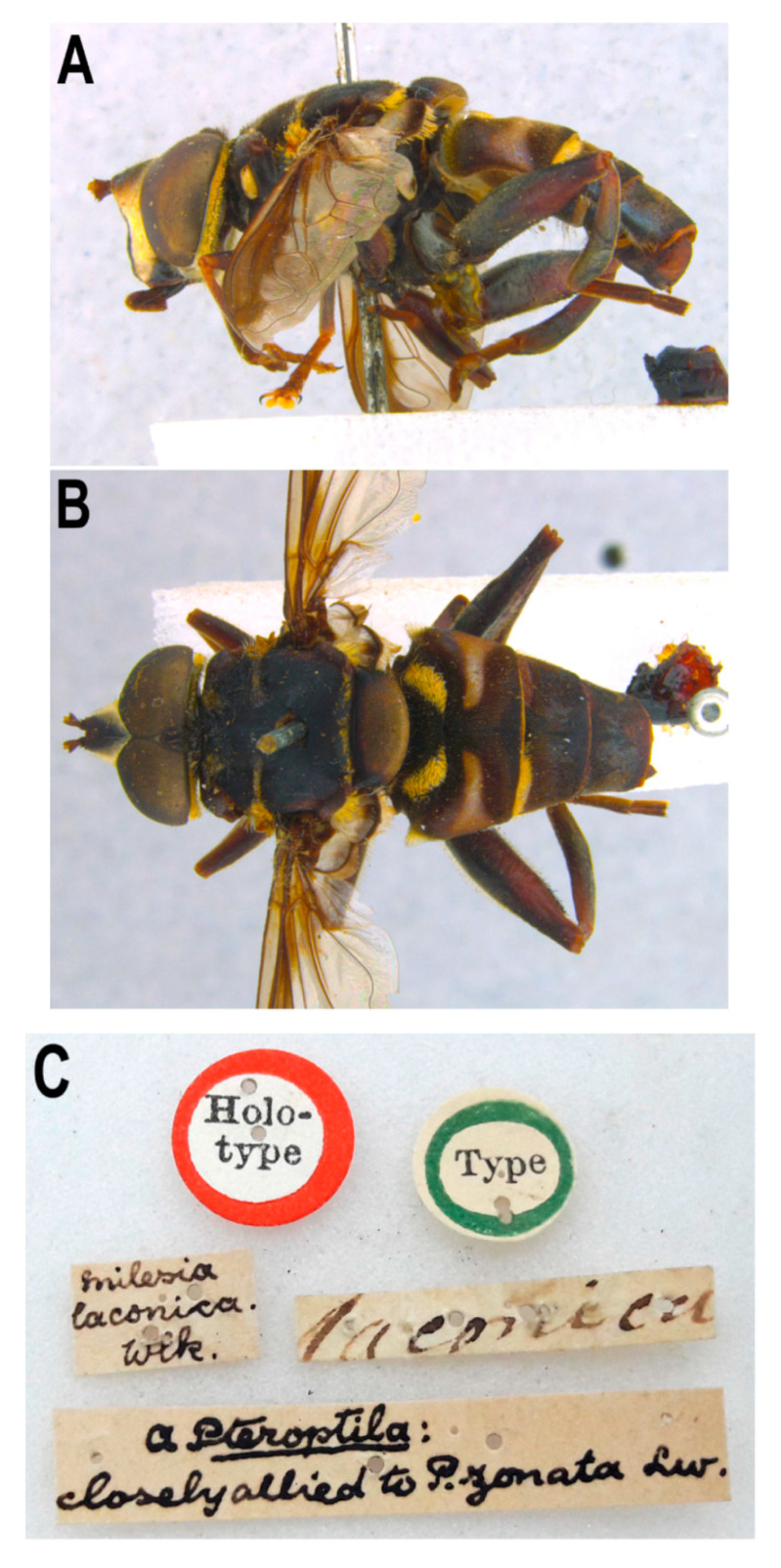
*Meromacrus laconicus*, adult, male holotype. (**A**): entire body, lateral view. (**B**): body, dorsal view. (**C**): specimen labels.

- Material examined. Holotype of *M. laconicus* (Figure 13): 1 male, Holotype (printed in a round label with red margin)/Type (printed in a round label with blue/green margin)/*Milesia laconica* Wlk. (hand written)/*laconica* (hand written)/a *Pteroptila*: closely allied to *P. zonata* Lw (hand written). Genitalia dissected and stored in a plastic microvial attached to the pin [NHM]; ***Mexico***: 15 males and 16 females (all except for 11 males and 9 females with puparia attached to the pin), (Yabucú (Acanceh), Yucatán, México), 20.81192, −89.41275, 15.03.2014, en *Ceiba pentandra*, oquedad en tronco podado (‘hole in a pollard’), leg.: J. Quinto, L 15.3.14, P: 22.3.14, A: 2.4.14, det. as Meromacron spm 8 by J. Quinto (1 male), L 15.3.14, P: 27.3.14, A: 8.4.14, det. as Meromacron spm 29 by J. Quinto (1 male), L 15.3.14, P: 2.4.14, A: 11.4.14, det. as Meromacron spm 39, 40 and 41 by J. Quinto (2 males and 1 female), L 15.3.14, P: 25.3.14, A: 6.4.14, det. as Meromacron spm 17, 19 and 21 by J. Quinto (1 male and 2 females), L 15.3.14, P: 25.3.14, A: 6.4.14, det. as Meromacron spm 18 by J. Quinto (1 male), L 15.3.14, P: 18.3.14, A: 1.4.14, det. as Meromacron spm 4 by J. Quinto (1 male), L 15.3.14, P: 31.3.14, A: 9.4.14, det. as Meromacron spm 31 by J. Quinto (1 male), L 15.3.14, P: 2.3.14, A: 7.4.14, det. as Meromacron spm 12 by J. Quinto (1 male), L 15.3.14, P: 26.3.14, A: 7.4.14, det. as Meromacron spm 22 and 26 by J. Quinto (2 males), L 15.3.14, P: 27.3.14, A: 7.4.14, det. as Meromacron spm 13 by J. Quinto (1 male), L 15.3.14, P: 31.3.14, A: 9.4.14, det. as Meromacron spm 32 and 35 by J. Quinto (2 males), L 15.3.14, P: 25.3.14, A: 4.4.14, det. as Meromacron spm 11 by J. Quinto (1 male), L 15.3.14, P: 31.3.14, A: 9.4.14, det. as Meromacron spm 33 by J. Quinto (1 male), L 15.3.14, P: 26.3.14, A: 7.4.14, det. as Meromacron spm 23, 24, 25 and 27 by J. Quinto (4 females), L 15.3.14, P: 7.4.14, A: 17.4.14, det. as Meromacron spm 45 by J. Quinto (1 female), L 15.3.14, P: 31.3.14, A: 11.4.14, det. as Meromacron spm 43 by J. Quinto (1 female), L 15.3.14, P: 8.4.14, A: 19.4.14, det. as Meromacron spm 46 by J. Quinto (1 female), L 15.3.14, P: 25.3.14, A: 6.4.14, det. as Meromacron spm 20 by J. Quinto (1 female), L 15.3.14, P: 11.4.14, A: 21.4.14, det. as Meromacron spm 48 and 49 by J. Quinto (2 females), L 15.3.14, P: 2.4.14, A: 12.4.14, det. as Meromacron spm 43 and 51 by J. Quinto (2 females), L 15.3.14, P: 28.3.14, A: 11.4.14, det. as Meromacron spm 42 by J. Quinto (1 female), L 15.3.14, P: 12.4.14, A: 24.4.14, det. as Meromacron spm 50 by J. Quinto (1 female); ***Costa Rica***: One male with puparium, CR12 (Volcán Tenorio, Alajuela, Upala), 2.3.06, leg. Mª Marcos García and G. Rotheray.
- Taxonomic notes. We have examined the male holotype of *M. laconicus* (Figure 13), including its genitalia in comparison with our specimens. In the holotype, the legs are darkened (Figure 13A,B) but in our specimens the legs are red, somewhat black apically in the femora. The shape of the cercus and surstylus in the holotype differs slightly from that found in the Mexican and Costa Rican specimens. In addition, the cercus and surstylus in the Mexican and Costa Rican material also displays certain variability in shape: the cercus can be round to trapezoidal, and the surstylus can be narrower or wider apically or even wedge-shaped apically. We consider this as intraspecific variability, since a specimen with holotype-like cercus (UA7ME) and two other specimens with different cercus shape (UA8ME and UA10ME) were shown to be conspecific in the COI gene tree (see Section 3.4.4).

#### 3.4.3. *Meromacrus ruficrus* (Wiedemann, 1830)

**Figure 14:**
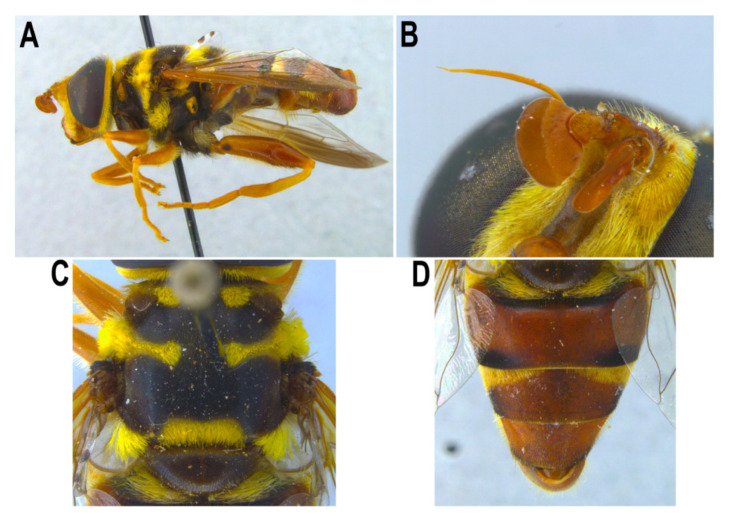
*Meromacrus ruficrus*, adult male. (**A**): entire body, lateral view. (**B**): right antenna, lateral view of inner side. (**C**): thorax, dorsal view. (**D**): abdomen, dorsal view.

Figure 2B and Figure 14

**Figure 15:**
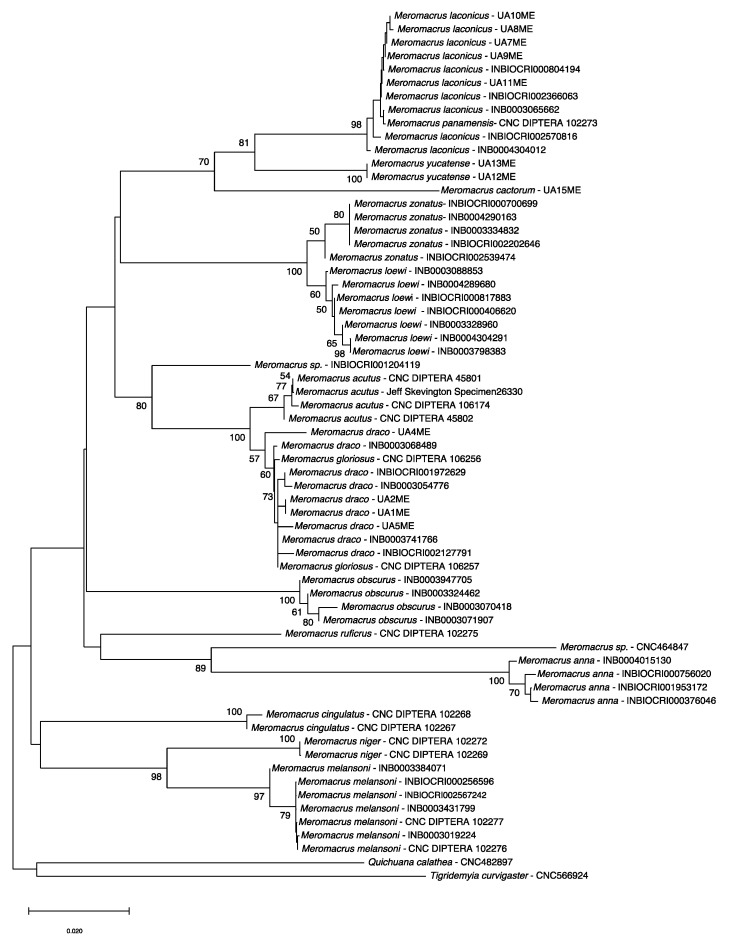
Neighbour-joining tree of all *Meromacrus* specimens genetically analysed using COI data. All data are available from the public dataset on BOLD: *Meromacrus* revision (DS-SYRMEROM). Synonyms: *Meromacrus draco* (= *Meromacrus gloriosus*), *Meromacrus panamensis* (= *Meromacrus laconicus*). Bootstrap supports over 50% are shown on the tree.

- Material examined. 1 male, Cuba, Habana del Este, Cerro de la Coca, 55m, 9-II-2001, leg. Mª A. Marcos García [CEUA].
- Taxonomic notes. Distinctive species due to the shape of basoflagellomere (Figure 14B), which is wider than long, and the male genitalia (Figure 2B). The CEUA specimen did not yield a genetic sequence, but another specimen from CNC did, and shows that this species clearly differs in COI from the other analysed species (Figure 15).

#### 3.4.4. Gene Trees and Pairwise Comparisons

Uncorrected pairwise genetic distances are presented in a table as Supplementary Material. Intraspecific variation ranged from 0.00 to 1.67% and averaged 0.25%. Interspecific variation ranged from 0.61% to 14.81% and averaged 8.76%. A neighbour joining tree is shown in Figure 15 and was used along with pairwise distances to explore taxonomic issues. Hypothesized relationships between *Meromacrus* species are illustrated on a maximum-likelihood gene tree (Supplementary Figure S1). Both trees show the two new species as independent clades (Figure 15 and Supplementary Figure S1), supporting the morphological species concepts. A CNC specimen labelled as *Meromacrus panamensis* grouped together with *M. laconicus*, while specimens labelled as *M. draco* and *M. gloriosus* grouped together in a separate clade (Figure 15). The position of *Meromacrus cingulatus* within the trees (Figure 15 and Supplementary Figure S1) is unresolved, with bootstrap values below 50%. Furthermore, *M. cingulatus* falls outside the *Meromacrus* clade in the ML tree (Supplementary Figure S1).

## 4. Discussion

After this study, which represents the first step towards a revision of the genus *Meromacrus* (which is being carried out by the authors of the present work), the number of valid species in this genus is 44. Two morphologically distinct species were described, *M. cactorum* sp. nov. and *M. yucantense* sp. nov., and *M. draco* was proposed as junior synonym of *M. gloriosus* on the basis of morphological and COI evidence. The status of two species genetically analysed in this paper (specimens CNC464847 and INBIOCRI001204119) is still pending of confirmation (Figure 15).

Adults and puparia of *M. cactorum* sp. nov. and *M. yucatense* sp. nov. differ considerably in morphology. Conspicuous differences can be found in the antenna shape (Figure 1D and Figure 7C), length of eye contiguity (Figure 1C and Figure 7B), size and density of yellow tomentose pile in thorax and abdomen (Figure 1B,E,F and Figure 7D,E), metafemur size (Figure 1A and Figure 7A), shape of male genitalia (Figure 2A,C), etc. Hull [20] grouped the *Meromacrus* species he studied according to the presence or absence of conspicuous markings of yellow to brownish tomentum. The two new species each agree with the characters of these two morphological groups (see new species descriptions). Hull [20] recognises that the dark or black-bodied species have brown pigmentation inside the loop of the vein R4+5, but our dark species, *M. cactorum* sp. nov., has the wing hyaline. We interpret that the hyaline loop is the natural state of this character in *M. cactorum* sp. nov., even though all studied specimens were reared. The two suggested morphological groups of Hull [20] appear to have no COI support. For example, *M. cactorum* sp. nov., with very slight markings of tomentum, clusters together in the ML tree with *M. laconicus* and *M. yucatense* sp. nov. (Supplementary Figure S1), both with obvious markings of tomentum. Further molecular markers and species should be analysed to test the phylogenetic significance of Hull’s putative groups.

*Meromacrus loewi* and *M. zonatus* are the closest taxa genetically (0.61–0.98% different) but cluster separately on the tree (Figure 15) and are morphologically distinctive. *Meromacrus zonatus* has golden-yellow tomentose pile on the head (occiput and frons), while *M. loewi* has not. In addition, *M. loewi* has less golden-yellow tomentose pile on the scutum, making the white-pollinose stripes of the scutum more visible than those of *M. zonatus*. *Meromacrus acutus* and *M. gloriosus* are also genetically close (0.47 to 2.29% different) but cluster separately (Figure 15) and are morphologically distinctive [9,20]. *Meromacrus draco* and *M. gloriosus* are interdigitated on the NJ tree (Figure 15) and have 0.00–1.57% pairwise differences; this supports the morphological justification presented above that these taxa are synonymous. Similarly, the single specimen of *M. panamensis* is nested within *M. laconicus* (Figure 15) and differs from them by 0.00–0.46%; this supports the morphological decision to synonymize these species made by Blatch et al. [4]. All other species have significant barcode gaps of more than 3% (see Supplementary material). The two new species, *M. cactorum* sp. nov. and *M. yucatense* sp. nov., are closely related to *M. laconicus* based on COI evidence (Supplementary Figure S1). Although *Meromacrus* is not monophyletic based on this analysis (*M. cingulatus* falls between the outgroup taxa, Supplementary Figure S1), a more comprehensive analysis using multiple markers is needed to confirm or refuse this preliminary result. Nonetheless, and suggesting the possible non-monophyly, *M. cingulatus* morphology differs from that of all other species represented in the COI-based trees, for example in having several yellow fasciae in the terga 3–4.

The puparia of *M. yucatense* sp. nov. and *M. cactorum* sp. nov. key out to *Meromacrus* in the key to the genera of Neotropical long-tailed syrphid larvae of Pérez-Bañón et al. [9], and they also have the shared characters stated for the *Meromacrus* species examined by these authors. *Meromacrus yucatense* sp. nov. anterior spiracles (Figure 9A) are clearly similar to those of the other known *Meromacrus* puparia, with all the respiratory openings arranged on a flat plate in the spiracle ventral surface. However, *M. cactorum* sp. nov. puparia have the respiratory openings of the anterior spiracles differently arranged, with paired openings on slightly protruding areas along the ventral curved spiracle surface (Figure 5A). In addition, the number of respiratory openings is clearly lower in *M. cactorum* sp. nov. than in other species, since *M. cactorum* sp. nov. has up to 10 openings while the known puparia of other *Meromacrus* species have at least double number of openings [9]. A higher number of respiratory openings might be an adaptation to live in aquatic media where the concentration of—diluted—oxygen is lower than in sites more exposed to the aerial media, such as the decaying cactus where *M. cactorum* sp. nov. was found. In the same way, the different characters found on the head skeletons of the two new species might be regarded as an indicator of their feeding media. *Meromacrus yucatense* sp. nov. does not have mandibular hooks while *M. cactorum* sp. nov. may use its mandibular hooks and its more sclerotised head skeleton to grasp firmer materials to obtain food rather than only filtering the fluid media as *M. yucatense* sp. nov.

Pupal spiracles of the two new species also look quite similar to those described previously of other species. *Meromacrus cactorum* sp. nov. and *M. yucatense* sp. nov. tubercle bands do not reach the ventral surface (Figure 5D and Figure 9D), separating these two species from *M. currani*, *M. draco* and *M. laconicus*. *Meromacrus cactorum* sp. nov. pupal spiracles taper apically (Figure 5D), as in *M. acutus*. However, while tubercle bands do not cover the entire length of the tube in *M. acutus*, they do almost entirely cover the surface of the spiracle in *M. cactorum* sp. nov. *Meromacrus yucatense* sp. nov. pupal spiracles do not taper apically (Figure 5C,D) as the ones mentioned before. Differing from *M. obscurus* and *M. laconicus*, *M. yucatense* sp. nov. does not have any longitudinal ridges nor ornaments on the ventral surface of the pupal spiracles (Figure 9D), which makes *M. yucatense* sp. nov. easily distinguishable. In addition, *M. yucatense* sp. nov. has small and scarce setae covering the surface of the bands of the pupal spiracles (Figure 9B), similarly to *M. draco* but in higher number than this species. This may be another evidence of the close relationship between the genera *Meromacrus* and *Habromyia*, as already indicated by Pérez-Bañón et al. [9]. *Habromyia coerulithorax* Williston, 1888 has a higher number of pupal spiracular setae than any described species of *Meromacrus*. However, *Meromacrus yucatense* sp. nov., in addition to *M. gloriosus*, seems to be another morphological intermediate between the presence and the absence of pupal spiracular setae.

The images presented in this paper show some significant differences in the PRP morphology of the two new species (Figure 5E,F and Figure 9E,F). The PRP of *M. cactorum* sp. nov. and *M. yucatense* sp. nov. are very different, especially in their shape and interspiracular setae. *M. cactorum* sp. nov. has subcylindrical to oval shaped PRP in cross section near its apical end, while *M. yucatense* sp. nov. has a strongly flattened oval-shaped perimeter along the entire PRP tube. Apart from this, the interspiracular setae along the perimeter of the spiracular plate show different forms. Those of *M. cactorum* sp. nov. are fan-looking and multibranched, but *M. yucatense* sp. nov. interspiracular setae are pectinate and have two morphotypes, one with two branches and the other uniramous. Further research on PRP SEM images might provide additional characters to separate larvae/puparia of other *Meromacrus* species such as those described in Pérez-Bañón et al. [9], who described only the anterior and/or pupal spiracles.

The number of *Meromacrus* species for which their early stages are known increases now to eight, i.e., 18% of described species in this genus. Our still-poor knowledge of *Meromacrus* larval biology involves a reasonably wide range of plants and breeding sites (see Introduction and Results) that suffice to anticipate an evolutionary history hypothesis strongly supported in the adaptation of larvae to novel breeding sites/plants, as in *Copestylum* [28,30,31] and *Quichuana* syrphids [32]. The three species reared from the same rotting stump in Yucatan, Mexico (*M. gloriosus*, *M. laconicus* and *M. yucatense* sp. nov.) are not all closely related (Supplementary Figure S1), suggesting that larval ecology is relatively consistent throughout the genus. Larvae of *M. yucatense* sp. nov. were found in the water-filled hole of a stump. Most known larvae of *Meromacrus* are also found in different kinds of water holes or pockets above ground level. However, larvae of *M. cactorum* sp. nov. were found in decaying parts of cacti. These are the first larvae of *Meromacrus* ever found in cacti, but not in decaying plant materials, since other larvae of this genus have been reported from banana stems and coffee pulp [9,12]; nonetheless, the species thought to be associated with banana stems (*M. gloriosus*, as *M. draco*) was actually collected from aground cavity filled with mud and incidentally containing a banana plant stem inside which its larva was found [4] and might not be then genuinely associated to banana stems. The putative high adaptability of *Meromacrus* larvae to breeding sites in different plants, together with this paper findings (two new species found as larvae in two sporadic sampling events) suggest the high number of *Meromacrus* species awaiting discovery in the New World. The findings as larvae of *M. cactorum* sp. nov. and *M. yucatense* sp. nov. also reinforce the idea of early stage sampling as an important method to inventory biodiversity and find out species requirements in the Neotropical ecosystems.

## 5. Conclusions

Morphological, genetic and biological evidence was combined from specimens deposited in different collections to shed light on the systematics of the New World genus *Meromacrus*. According to the objectives of the present study, conclusions are as follows:

(a) *Meromacrus* taxonomy was partly revised, with two species new to science (*M. cactorum* sp. nov. and *M. yucatense* sp. nov.) and *M. draco* being synonymised under *M. gloriosus*. The male genitalia of *M. ruficrus* was figured for the first time to facilitate its unequivocal identification based on genitalia characters.
(b) The two new species were reared from saprophagous larvae collected in rot-holes (*M. yucatense* sp. nov.) and decaying cacti (*M. cactorum* sp. nov.), representing the first *Meromacrus* larva ever found in cacti. Larvae of *M. cactorum* sp. nov. appear to have specific morphological adaptations to their breeding site and substrate, while those of *M. yucatense* sp. nov. have a morphology most similar to that of other *Meromacrus* species.
(c) The existing identification key to *Meromacrus* puparia was further completed with the addition of the two new species’ puparia. With these additions, the utility of this key increases and diversity surveys based on early stages become even more feasible than prior to this study.
(d) A NJ tree—with 16 named and unnamed taxa putatively assigned to the genus *Meromacrus*—compiling all COI data available to authors of the present paper was produced to show how the new species clearly diverge from other named species and to support the proposed synonymy.

In summary, this study becomes the first step towards a taxonomic, biological and phylogenetic revision of the genus *Meromacrus*, in such a way these flies can be used in future as bioindicators and models of adaptive radiations.

## Figures and Tables

**Table 1:** Custom primers used in the DNA analysis of *Meromacrus* adults (Diptera: Syrphidae).

Primer Name	Primer Design	Primer Sequence
Heb-F	Folmer [22]	GGT CAA CAA ATC ATA AAG ATA TTG G
COI-Fx-A-R	Kelso (unpublished data) ^1^	CGD GGR AAD GCY ATR TCD GG
COI-Fx-B-F	Kelso (unpublished data) ^1^	GGD KCH CCN GAY ATR GC
COI-Fx-B-R	Kelso (unpublished data) ^1^	GWA ATR AAR TTW ACD GCH CC
COI-Fx-C-F	Kelso (unpublished data) ^1^	GGD ATW TCH TCH ATY YTA GG
COI-780R	Gibson [23]	CCA AAA AAT CAR AAT ARR TGY TG

^1^ Unpublished procedure.

**Table 2:** *Meromacrus* and outgroup specimens used for DNA barcode analysis. All data are available from the public dataset on BOLD: *Meromacrus* revision (DS-SYRMEROM).

Species	Sample ID	Deposition	Country	GenBank Number
*M. acutus*	CNC DIPTERA 106174	CNC	USA	MK585702
*M. acutus*	CNC DIPTERA 45801	CNC	USA	MK585689
*M. acutus*	CNC DIPTERA 45802	CNC	USA	MK585707
*M. acutus*	Jeff_Skevington_Specimen26330	CNC	USA	MK585690
*M. anna*	INB0004015130	INBIO	Costa Rica	MN621091
*M. anna*	INBIOCRI000376046	INBIO	Costa Rica	MN621114
*M. anna*	INBIOCRI000756020	INBIO	Costa Rica	MN621092
*M. anna*	INBIOCRI001953172	INBIO	Costa Rica	MN621081
*M. cactorum*	UA15ME	CIBIO	Peru	MK585699
*M. cingulatus*	CNC DIPTERA 102267	CNC	Argentina	MK585705
*M. cingulatus*	CNC DIPTERA 102268	CNC	Brazil	MK585693
*M. gloriosus*	CNC DIPTERA 106256	CNC	USA	MK585710
*M. gloriosus*	CNC DIPTERA 106257	CNC	USA	MK585692
*M. gloriosus*	INB0003054776	INBIO	Costa Rica	MN621079
*M. gloriosus*	INB0003068489	INBIO	Costa Rica	MN621077
*M. gloriosus*	INB0003741766	INBIO	Costa Rica	MN621080
*M. gloriosus*	INBIOCRI001972629	INBIO	Costa Rica	MN621110
*M. gloriosus*	INBIOCRI002127791	INBIO	Costa Rica	MN621109
*M. gloriosus*	UA1ME	CEUA	Mexico	MK585708
*M. gloriosus*	UA2ME	CIBIO	Mexico	MK585694
*M. gloriosus*	UA4ME	CEUA	Costa Rica	MN621104
*M. gloriosus*	UA5ME	CIBIO	Costa Rica	MK585691
*M. laconicus*	CNC DIPTERA 102273	CNC	Brazil	MK585698
*M. laconicus*	INB0003065662	INBIO	Costa Rica	MN621099
*M. laconicus*	INB0004304012	INBIO	Costa Rica	MN621095
*M. laconicus*	INBIOCRI000804194	INBIO	Costa Rica	MN621094
*M. laconicus*	INBIOCRI002366063	INBIO	Costa Rica	MN621083
*M. laconicus*	INBIOCRI002570816	INBIO	Costa Rica	MN621102
*M. laconicus*	UA10ME	CIBIO	Mexico	MK585706
*M. laconicus*	UA11ME	CIBIO	Mexico	MK585703
*M. laconicus*	UA7ME	CIBIO	Mexico	MK585684
*M. laconicus*	UA8ME	CIBIO	Mexico	MK585688
*M. laconicus*	UA9ME	CIBIO	Mexico	MK585695
*M. loewi*	INB0003088853	INBIO	Costa Rica	MN621105
*M. loewi*	INB0003328960	INBIO	Costa Rica	MN621098
*M. loewi*	INB0004289680	INBIO	Costa Rica	MN621085
*M. loewi*	INB0004304291	INBIO	Costa Rica	MN621089
*M. loewi*	INBIOCRI000406620	INBIO	Costa Rica	MN621106
*M. loewi*	INBIOCRI000817883	INBIO	Costa Rica	MN621093
*M. lowei*	INB0003798383	INBIO	Costa Rica	MN621078
*M. melansoni*	CNC DIPTERA 102276	CNC	Costa Rica	MK585697
*M. melansoni*	CNC DIPTERA 102277	CNC	Costa Rica	MK585681
*M. melansoni*	INB0003019224	INBIO	Costa Rica	MN621111
*M. melansoni*	INB0003384071	INBIO	Costa Rica	MN621082
*M. melansoni*	INB0003431799	INBIO	Costa Rica	MN621087
*M. melansoni*	INBIOCRI000256596	INBIO	Costa Rica	MN621088
*M. melansoni*	INBIOCRI002567242	INBIO	Costa Rica	MN621113
*M. niger*	CNC DIPTERA 102269	CNC	Argentina	MK585683
*M. niger*	CNC DIPTERA 102272	CNC	Argentina	MK585686
*M. obscurus*	INB0003070418	INBIO	Costa Rica	MN621112
*M. obscurus*	INB0003071907	INBIO	Costa Rica	MN621103
*M. obscurus*	INB0003324462	INBIO	Costa Rica	MN621090
*M. obscurus*	INB0003947705	INBIO	Costa Rica	MN621076
*M. ruficrus*	CNC DIPTERA 102275	CNC	Bahamas	MK585687
*M.* sp.	CNC464847	CNC	Peru	MK585696
*M.* sp.	INBIOCRI001204119	INBIO	Costa Rica	MN621107
*M. yucatense*	UA12ME	CIBIO	Mexico	MK585685
*M. yucatense*	UA13ME	CIBIO	Mexico	MK585682
*M. zonatus*	INB0003334832	INBIO	Costa Rica	MN621101
*M. zonatus*	INB0004290163	INBIO	Costa Rica	MN621096
*M. zonatus*	INBIOCRI000700699	INBIO	Costa Rica	MN621084
*M. zonatus*	INBIOCRI002202646	INBIO	Costa Rica	MN621108
*M. zonatus*	INBIOCRI002539474	INBIO	Costa Rica	MN621086
*Tigridemyia curvigaster*	CNC566924	CNC	Taiwan	MN621097
*Quichuana calathea*	CNC482897	CNC	Ecuador	MN621100

## Supplementary Materials

The following are available online at https://www.mdpi.com/2075-4450/11/11/791/s1, Figure S1: Maximum-likelihood COI gene tree for the hypothesized relationships between analysed *Meromacrus* species. Bootstrap supports over 50% are shown on the tree; Table S1: uncorrected pairwise genetic distances (p-distance) in *Meromacrus* syrphids.
