# Supplementary material for: Morphological, Genetic and Biological Evidences to Understand Meromacrus Rondani Diversity: New Species and Early Stages (Diptera: Syrphidae)
Source: Insects. 2020 Nov 12;11(11):791. doi: 10.3390/insects11110791 (PMC7698139; doi:10.3390/insects11110791)

Supplementary material 1. Maximum-likelihood COI gene tree for the hypothesized relationships between analysed *Meromacrus* species. Bootstrap supports over 50% are shown on the tree.

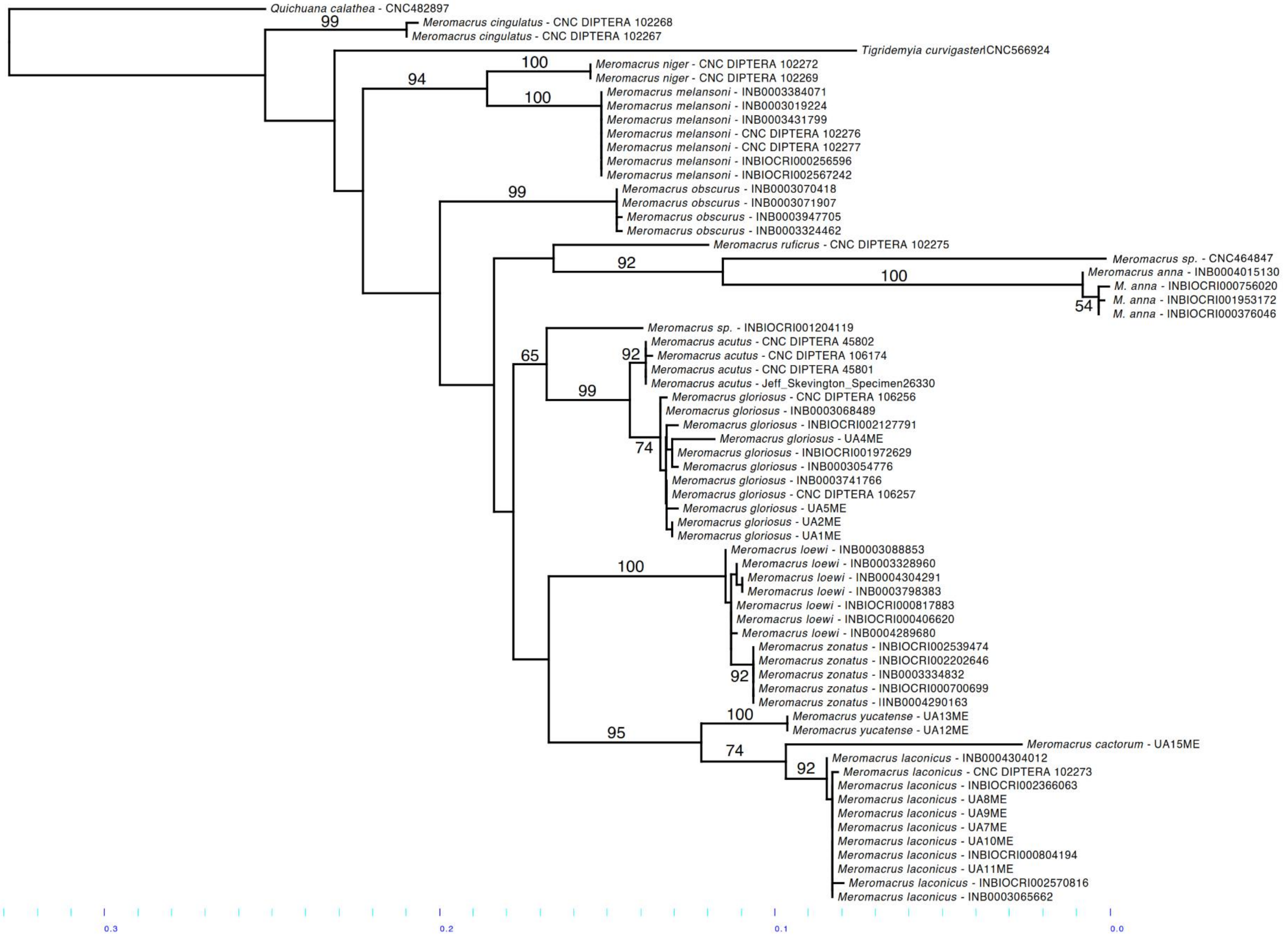

Supplement: Supplementary file 1 [file insects-11-00791-s001.zip › MEROMACRUS SUPPL MAT ROUND 2/Supplementary material 1.pdf]
